# NS5-V372A and NS5-H386Y variations are responsible for differences in interferon α/β induction and co-contribute to the replication advantage of Japanese encephalitis virus genotype I over genotype III in ducklings

**DOI:** 10.1371/journal.ppat.1008773

**Published:** 2020-09-03

**Authors:** Chenxi Li, Di Di, Hui Huang, Xin Wang, Qiqi Xia, Xiaochun Ma, Ke Liu, Beibei Li, Donghua Shao, Yafeng Qiu, Zongjie Li, Jianchao Wei, Zhiyong Ma

**Affiliations:** Shanghai Veterinary Research Institute, Chinese Academy of Agricultural Science, Shanghai, P.R. China; National Institute of Allergy and Infectious Diseases, UNITED STATES

## Abstract

Japanese encephalitis virus (JEV) genotype I (GI) replicates more efficiently than genotype III (GIII) in birds, and this difference is considered to be one of the reasons for the JEV genotype shift. In this study, we utilized duck embryo fibroblasts and domestic ducklings as *in vitro* and *in vivo* models of a JEV amplifying avian host to identify the viral determinants of the differing replication efficiency between the GI and GIII strains in birds. GI strains induced significantly lower levels of interferon (IFN)-α and β production than GIII strains, an effect orrelated with the enhanced replication efficiency of GI strains over GIII strains. By using a series of chimeric viruses with exchange of viral structural and non-structural (NS) proteins, we identified NS5 as the viral determinant of the differences in IFN-α and β induction and replication efficiency between the GI and III strains. NS5 inhibited IFN-α and β production induced by poly(I:C) stimulation and harbored 11 amino acid variations, of which the NS5-V372A and NS5-H386Y variations were identified to co-contribute to the differences in IFN-α and β induction and replication efficiency between the strains. The NS5-V372A and NS5-H386Y variations resulted in alterations in the number of hydrogen bonds formed with neighboring residues, which were associated with the different ability of the GI and GIII strains to inhibit IFN-α and β production. Our findings indicated that the NS5-V372A and NS5-H386Y variations enabled GI strains to inhibit IFN-α and β production more efficiently than GIII strains for antagonism of the IFN-I mediated antiviral response, thereby leading to the replication and host adaption advantages of GI strains over GIII strains in birds. These findings provide new insight into the molecular basis of the JEV genotype shift.

## Author summary

The Japanese encephalitis virus (JEV) transmission cycle is maintained by mosquitoes and amplification hosts (pigs and birds). In areas without large pig populations, birds play a major role in the maintenance of the JEV transmission cycle. The shift in the dominant JEV genotype from genotype III (GIII) to genotype I (GI) is occurring in most countries in Asia. GI strains replicates more efficiently than GIII strains in birds, and this difference has been considered one of the reasons for the JEV genotype shift. By using a series of chimeric viruses with exchange of viral structural and non-structural (NS) proteins, we demonstrated that NS5 is the viral determinant of the differences in replication efficiencies between the GI and III strains in birds. Furthermore, the NS5-V372A and NS5-H386Y variations were identified to co-contribute to the differences in type I interferon (IFN-I) induction and replication efficiency between the strains. Our findings suggested that the NS5-V372A and NS5-H386Y variations enable GI strains to inhibit IFN-I production more efficiently than GIII strains, thus resulting in antagonism of the IFN-I mediated antiviral response and consequently conferring a replication and host adaption advantage to GI strains over GIII strains in birds. These findings provide new insight into the molecular basis of the JEV genotype shift.

## Introduction

Japanese encephalitis virus (JEV) belongs to the genus *Flavivirus* of the family *Flaviviridae*, which comprises more than 70 species—including West Nile virus (WNV), dengue virus (DENV), Zika virus (ZIKV), yellow fever virus, and tick-borne encephalitis virus—and causes Japanese encephalitis in humans and reproductive disorders in pigs[1, 2]. The annual incidence of human cases caused by JEV has been reported to be in the range of 30,000 to 50,000, with approximately 10,000 to 15,000 fatalities[3]. JEV was first isolated in Japan in 1935 and is currently prevalent mainly in East and Southeast Asia[3–5]. To date, JEV is considered to extend to new geographic regions, thus posing a risk to public health worldwide[6, 7].

The JEV genome is a single-stranded, positive-sense RNA approximately 11 kb in length that encodes a single polyprotein. After translation, the polyprotein is cleaved by both host and viral proteases into three structural proteins (capsid I, pre-membrane/membrane (prM), and envelopeI) and seven non-structural proteins (NS1, NS2A, NS2B, NS3, NS4A, NS4B, and NS5)[8]. According to the nucleotide sequence of the E gene, JEV is phylogenetically classified into five genotypes (genotype I to V), and most isolates belong to genotype I (GI) or genotype III (GIII)[9]. GIII was the dominant genotype from 1935 through the end of the 20^th^ century throughout most countries in Asia[10, 11]. GI was first identified in Cambodia in 1967 and then remained undetectable until a new strain re-emerged in China in 1979[12, 13]. Notably, the number of GI isolates has increased in the past 20 years, thus resulting in a JEV genotype shift from GIII to GI; the re-emergent GI has become the dominant genotype instead of GIII in most countries in Asia[14].

JEV is a mosquito-borne virus with a zoonotic transmission cycle maintained by mosquito vectors and vertebrate amplifying hosts (birds and pigs)[15]. As amplifying hosts of JEV, infected birds and pigs develop a sufficiently high viremia to infect mosquitoes, thereby playing a critical role in the maintenance and transmission of the JEV in nature[1, 16, 17]. Although pigs are essential for JEV amplification, some epidemics occur in areas with low pig populations[18, 19]. Moreover, the JEV genotype shift has occurred in some endemic countries where pig-breeding is not common, such as Korea[18], Malaysia[20], and India[21]. These observations suggest an essential contribution of birds to the maintenance and transmission of the JEV as well as the JEV genotype shift. Indeed, GI strains have been demonstrated to replicate more efficiently than GIII strains in domestic ducklings and young chickens, thus showing an advantage of GI strains over GIII strains in replication efficiency and host adaption in birds, and contributing to the JEV genotype shift [22, 23]. The mechanisms underlying this advantage are not known. A resent publication has demonstrated that three amino acid substitutions in NS2B/NS3 contribute to the replication advantage of GI strains over GIII strains in pigs and poultry[22], thus providing insight into the molecular basis of the JEV genotype shift. However, this molecular basis still remains largely unknown.

Type I interferon (IFN-I) serves as the first line host defense against flavivirus infection[24]. The ability to antagonize the IFN-I mediated antiviral response is a limiting factor associated with the replication efficiency and host adaptability of flavivirus[13, 14], thereby determining the species range of viral hosts[25]. For example, ZIKV antagonizes interferon-β (IFN-β) induction via an evolutionary NS1 mutation, thereby promoting replication efficiency in mosquitoes and enhancing neurovirulence in mammals[26]. A single amino acid substitution in NS4B of DENV enhances virus growth and fitness in human cells *in vitro* through an IFN-dependent host response[27]. For JEV, the replication efficiency and virulence positively correlate with the ability to inhibit the IFN-I mediated antiviral response[28]. GI strains replicate more efficiently than GIII strains in IFN-competent chicken-derived DF-1 cells but not in IFN-deficient Vero cells[22], thus implying that the ability to antagonize the IFN-I mediated antiviral response may determine the differences in replication efficiency between the GI and GIII strains in birds. In this study, we examined the differences in IFN-I induction between these strains by using duck embryo fibroblasts (DEF) and domestic ducklings as *in vitro* and *in vivo* avian models, respectively, and identified the viral determinant of the differences in replication efficiency between these strains in birds.

## Results

### GI strains induce lower levels of IFN-α and β production than GIII strains in duck-derived cells, but not in pig- and mouse-derived cells

**Fig 1:**
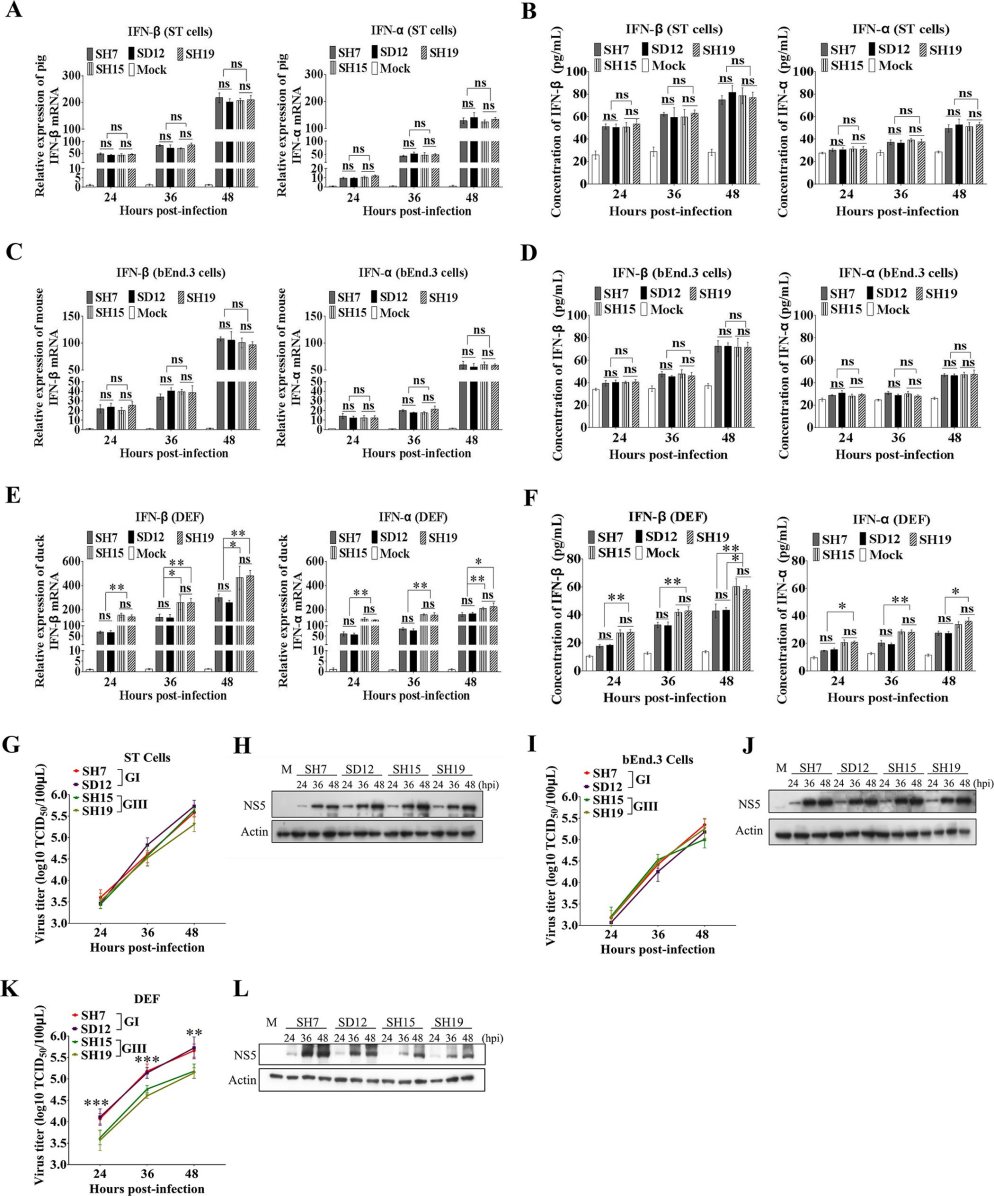
Differences in IFN-α and β induction and replication efficiency between the GI and GIII strains. ST, bEnd.3, and DEF cells were infected with GI (SH7 and SD12) and GIII (SH15 and SH19) strains at 0.1 MOI and harvested at 24, 36, and 48 hpi for measurement of IFN-α and β production and viral replication. (A, C and E) The mRNA levels of IFN-α and β in the cell pellets were examined by qRT-PCR. (B, D and F) The concentrations of IFN-α and β proteins in the supernatants were determined by ELISA. (G, I and K) The replication titers of GI and GIII strains in the supernatants were titrated with TCID_50_ assays in BHK cells and the significant differences between the average titers of GI and GIII strains were tested at each time point. (H, J and L) The levels of viral NS5 were examined with western blotting with anti-NS5 antibodies. All data are presented as mean ± SD from three independent experiments. ***, *p* < 0.001; **, *p* < 0.01; ns, no significant difference, by Student’s *t*-test.

The ability to antagonize the IFN-I mediated antiviral response has been speculated to determine the differences in replication efficiency between the GI and GIII strains in birds[22]. To test this possibility, we used two GI strains (SH7 and SD12) and two GIII strains (SH15 and SH19), all of which were isolated during 2015–2016[29], to infect cells derived from three types of JEV hosts: DEF, a swine testicular cell line (ST), and a mouse endothelial brain cell line (bEnd.3). We then analyzed the induction of IFN-α and β expression. The infected cells were harvested at 24, 36, and 48 hours post-infection (hpi) and briefly centrifuged to separate the cell pellets and supernatants. We then performed analysis of IFN-α and β expression at the mRNA level through quantitative real-time RT-PCR (qRT-PCR) and at the protein level through enzyme-linked immunosorbent assay (ELISA) (Fig 1). The viral replication was monitored on the basis of the 50% tissue culture infective dose (TCID_50_), western blotting (Fig 1), and immunofluorescence assays (S1 Fig). No significant differences in IFN-α and β induction at both the mRNA and protein levels between the GI and GIII strains were observed in either (Fig 1A and 1B) or bEnd.3 cells (Fig 1C and 1D); however, significant differences were observed in DEF (Fig 1E and 1F). The relative mRNA levels of IFN-α and β in GI-infected DEF were significantly lower than those in GIII-infected DEF (Fig 1E). Analysis of the concentrations of IFN-α and β protein in the supernatants revealed that GI strains induced markedly lower levels of IFN-α and β production than GIII strains (Fig 1F), thereby counteracting the host antiviral immune response in DEF, but not in ST and bEnd.3 cells, in a species-specific manner. To confirm that this result was species-specific but not cell type-specific, duck embryo kidney cells (DEK), a porcine iliac endothelium cell line (PIEC), and a mouse embryonic fibroblast cell line (MEF) were infected with the GI and GIII strains and the induction of IFN-α and β expression was analyzed. No significant differences in IFN-α and β induction between the GI and GIII strains were observed in either PIEC (S2A Fig) or MEF (S2B Fig); however, significant differences were observed in DEK (S2C and S2D Fig). These data further confirmed that the differences in IFN-α and β induction between the GI and GIII strains were species-specific.

The ability to counteract the IFN-I mediated antiviral response is orrelated with the replication efficiency of flavivirus[30, 31]. We therefore measured the replication levels of GI and GIII strains in infected cells at 24, 36, and 48 hpi. No significant differences in viral replication, as monitored by TCID_50_ assays and western blotting between the GI and GIII strains, were observed in ST cells (Fig 1G and 1H) or bEnd.3 cells (Fig 1I and 1J), in which the levels of IFN-α and β production were similar between the strains (Fig 1A, 1B, 1C and 1D). However, significant differences in viral replication between the GI and GIII strains were detected in DEF (Fig 1K and 1L), and the levels of IFN-α and β production significantly differed between the strains (Fig 1E and 1F). GI strains produced viral titers 0.5–1 log higher than GIII strains in DEF at 24, 36, and 48 hpi (Fig 1K). The levels of viral NS5 protein in GI-infected DEF were also notably higher than those in GIII-infected cells (Fig 1L). The replication levels of the GI and GIII strains were further measured in PIEC (S2E and S2F Fig), MEF (S2G and S2H Fig) and DEK (S2I and S2J Fig), significant differences in viral replication between the GI and GIII strains were detected in DEK, but not in PIEC and MEF. Overall, these data indicated that the replication efficiency of GI and GIII strains was associated with the level of IFN-α and β production, and the high replication titers of GI strains orrelated with low levels of IFN-α and β production.

Although mosquitoes are not considered to play a major role in the JEV genotype shift[22, 23, 32], we measured the replication levels of the GI and GIII strains in a mosquito-derived cell line (C6/36) and observed no significant differences in viral replication between the GI and GIII strains, as monitored by TCID_50_ assays (S2K Fig) and western blotting (S2L Fig), in agreement with the previous observations [22, 23].

### IFN-α and β determine the differences in replication efficiency between the GI and GIII strains in DEF

**Fig 2:**
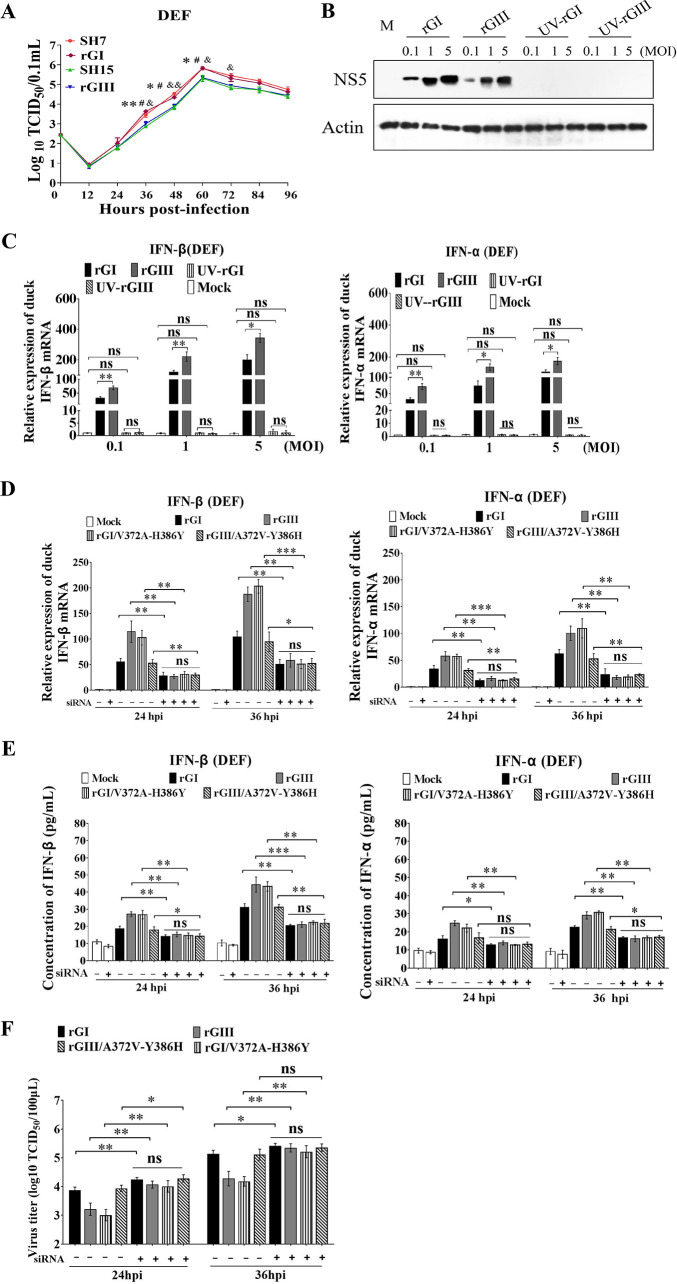
Replication of rGI and rGIII in IFN-α and β knockdown DEF. (A) DEF were infected with SH7, rGI, SH15 and rGIII at 0.1 MOI and harvested at the indicated time points. The replication titers in the supernatants were titrated with TCID_50_ assays in BHK cells and tested by Student’s *t*-test. The significant differences between rGI and rGIII at different time points are labeled (**, *p* < 0.01; *, *p* < 0.05). The significant difference between rGI and SH15 at different time points is marked (#, *p* < 0.05). The significant difference between rGIII and SH7 at different time points is indicated (&&, *p*<0.01; &, *p* < 0.05). (B and C) DEF were infected with rGI, UV-rGI, rGIII and UV-rGIII at 0.1, 1 and 5 MOI and harvested at 24 hpi for detection of NS5 levels by western blotting with anti-NS5 antibodies (B) and for measurement of IFN-α and β production at mRNA level by qRT-PCR (C). (D to F) DEF were treated with siRNA for silencing IFN-α or β expression (siRNA+), or with scrambled RNA control (siRNA-) and incubated for 12 h. The transfectants were subsequently infected with rGI, rGIII, rGI/V372A-H386Y and rGIII/A372V-Y386H at 0.1 MOI and harvested at 24 and 36 h for analysis of IFN-α and β induction at the mRNA levels by qRT-PCR (D) and protein levels by ELISA I as well as for measurement of replication titers by TCID_50_ assays in BHK cells (F). All data are presented as mean ± SD from three independent experiments. ***, *p* < 0.001; **, *p* < 0.01; *, *p* < 0.05; ns, no significant difference, by Student’s *t*-test.

The replication efficiency of GI and GIII strains was associated with the levels of IFN-α and β production (Fig 1), we therefore examined whether IFN-α and β determined the differences in replication efficiency between the GI and GIII strains in DEF. To this end, we chose the SH7 and SH15 strains as representative GI and GIII viruses, respectively, and constructed an infectious cDNA clone of each (denoted rGI for SH7 and rGIII for SH15) that was used as backbone for generation of chimeric recombinant viruses in subsequent experiments. Analysis of the replication kinetics in DEF revealed that rGI and rGIII exhibited virus titers similar to those of SH7 and SH15, respectively (Fig 2A).

We examined whether JEV replication was essential for IFN-α and β induction. rGI and rGIII were inactivated by ultraviolet (UV) irradiation and subsequently subjected to infection of DEF. DEF were inoculated with rGI, UV-inactivated-rGI (UV-rGI), rGIII, and UV-inactivated rGIII (UV-rGIII), and the levels of IFN-α and β expression were measured by qRT-PCR. In contrast to the cells infected with rGI and rGIII, no NS5 protein was detected in the cells infected with UV-rGI or UV-rGIII (Fig 2B), indicating that both UV-rGI and UV-rGIII were inactivated and unable to replicate in DEF. Analysis of IFN-α and β expression indicated that rGI and rGIII induced both IFN-α and β expression, with significant differences in the mRNA levels between rGI and rGIII. However, UV-rGI and UV-rGIII failed to induce IFN-α and β expression (Fig 2C). The levels of IFN-α and β expression in the cells infected with UV-rGI and UV-rGIII were similar to the basal levels of IFN-α and β expression in mock-infected cells. These results indicated that viral replication was essential for IFN-α and β induction.

To test whether IFN-α and β determined the differences in replication efficiency between the GI and GIII strains, DEF was treated with small interfering RNA (siRNA) to knock down IFN-α and β expression by RNA interference and subsequently infected with rGI and rGIII. In response to rGI and rGIII infection, the levels of IFN-α and β induction increased at the mRNA and protein levels in the cells treated with siRNA (siRNA+), as compared with mock-infected cells, but were significantly lower than those in the cells treated with scrambled RNA control (siRNA-) (Fig 2D and 2E), suggesting that IFN-α and β expression was silenced. Noticeably, no significant differences in IFN-α and β induction between rGI and rGIII were observed in the cells treated with siRNA (siRNA+) in the presence of rGI and rGIII infection (Fig 2D and 2E). Analysis of viral replication indicated that significant increases in replication titers of rGI and rGIII were observed in the cells treated with siRNA (siRNA+), as compared with the cells treated with scrambled RNA control (siRNA-) (Fig 2F). However, no significant differences in virus titers between rGI and rGIII were detected in the cells treated with siRNA (siRNA+) (Fig 2F). Overall, these observations demonstrated that JEV replication was essential for IFN-α and β induction, and IFN-α and β determined the differences in replication efficiency between the GI and GIII strains in DEF.

### Amino acid variations between the GI and GIII strains

Mutations in amino acid sequences alter the ability of flavivirus to induce of IFN-I production[26, 28]. To determine the viral determinants of the differences in IFN-α and β induction, we compared the amino acid sequences between the GI and GIII strains. A total of 50 representative GI and GIII strains, including the strains used in this study (S1 Table), were downloaded from the GenBank database and subjected to phylogenetic analysis and sequence alignment. The sequence alignment revealed a total of 43 amino acid variations with high conservation rates of 90%–100% and ten amino acid variations with relatively low conservation rates of 50%–89% in their respective genotypes between the GI and GIII strains (Table 1 and S3 Fig). The amino acid variations were distributed in three structural proteins (C, PrM, and E) and seven non-structural proteins (NS1, NS2A, NS2B, NS3, NS4A, NS4B, and NS5) (Table 1). Notably, some non-structural proteins with relatively high numbers of amino acid variations, such as NS1, NS3, and NS5, have been demonstrated to be involved in antagonizing the IFN-I mediated antiviral response in flavivirus[33–36].

**Table 1:** Amino acid variations conserved between GI and GIII strains.

Protein	GI residue/position/GIII residue										
C	R70K^a^	K100R^a^	S110G^a^	I120V^a^	T122I^a^						
PrM	A57T^a^	V58M^a^	**A140V(I)**^**b**^	S149N^a^							
E	M129T^a^	S222A^a^	T327S^a^	S366A^a^							
NS1	Q51K^a^	S70A^a^	R147H^a^	**N(D)175S**^**b**^	**L206Y(F)**^**b**^	R251K^a^	I298V^a^				
NS2A	I6V^a^	A97T^a^	T149S^a^	A151T^a^	R187K^a^						
NS2B	D55E^a^	E65D^a^	L99V^a^								
NS3	L14S^a^	S78A^a^	P105A^a^	**I(V)123V**^**b**^	D177E^a^	**S182N(T**)^**b**^	K185R^a^	D354E^a^			
NS4A	V110I^a^										
NS4B	**R(K)20K**^**b**^	**P(S)24S(P)**^**b**^	S73N^a^	V118A^a^							
NS5	D22E^a^	K101R^a^	R280K^a^	**R(K)287K**^**b**^	V372A^a^	**H386Y(H)**^**b**^	G429D^a^	L432R^a^	N438D^a^	G588E^a^	I878V^a^

a Amino acid variation with conservation rate of 90%-100% in GI and GIII strains.

b Amino acid variation with conservation rate of 50%-89% in GI and GIII strains and are highlighted in boldface.

### NS5 is the viral determinant of differences in IFN-α and β induction between the GI and GIII strains

**Fig 3:**
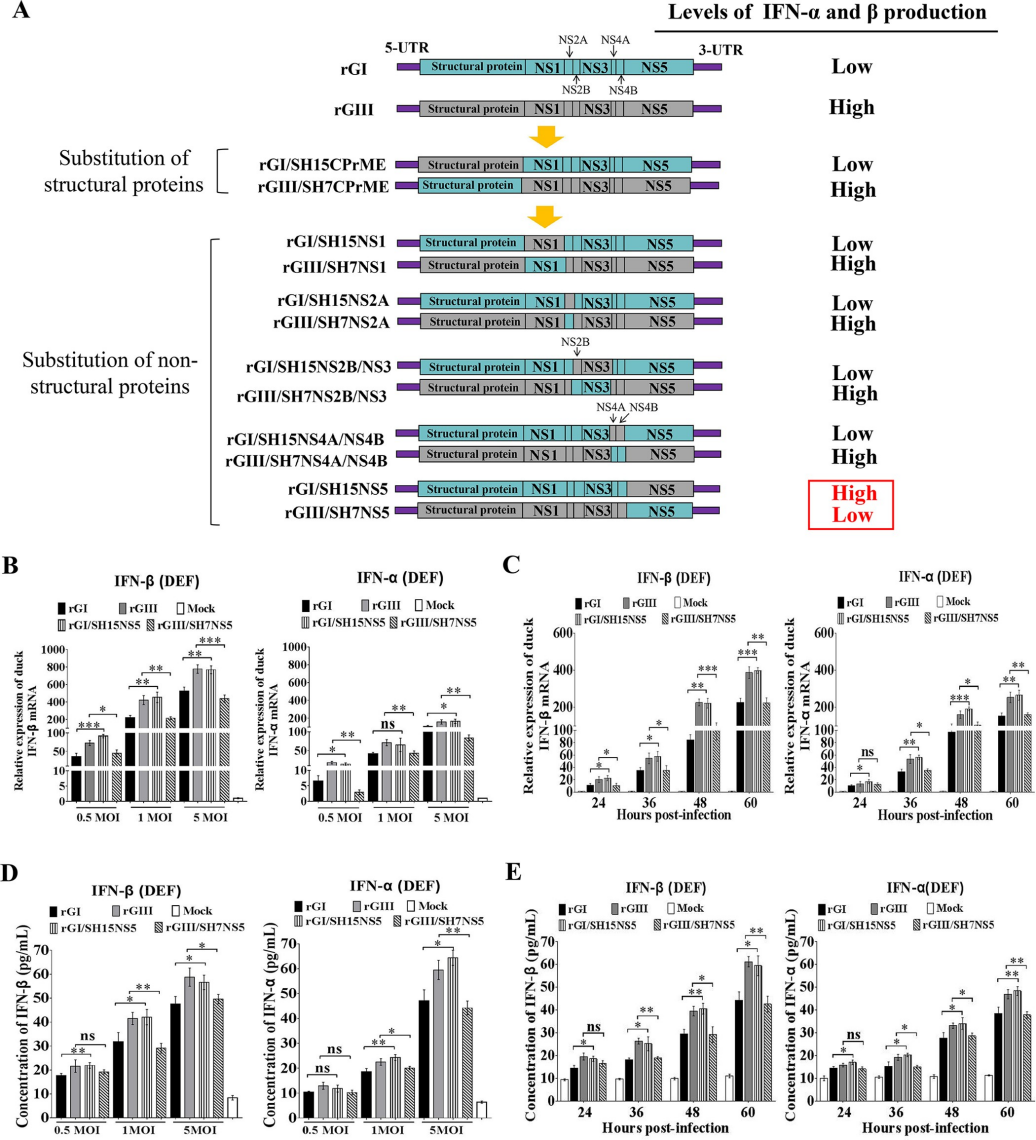
Identification of the viral determinant of different IFN-α and β induction. (A) Schematic diagram of parental and chimeric recombinant viruses used in this study. Viral proteins of GI and GIII strains are highlighted in blue and gray, respectively. (B and D) DEF were infected with the indicated recombinant viruses at a MOI of 0.5, 1, and 5 and harvested at 24 hpi for measurement of IFN-α and β production. (C and E) DEF were infected with the indicated recombinant viruses at 1 MOI and harvested at the indicated time points for measurement of IFN-α and β production. The mRNA levels of IFN-α and β in the cell pellets were examined by using qRT-PCR (B and C). The concentrations of IFN-α and β proteins in the supernatants were determined with ELISA (D and E). All data are presented as mean ± SD from three independent experiments. ***, *p* < 0.001; **, *p* < 0.01; *, *p* < 0.05; ns, no significant difference, by Student’s *t*-test.

GI strains induced lower levels of IFN-α and β production than GIII strains in duck-derived cells (Figs 1 and S2). We therefore examined which viral protein harboring the amino acid variations determined the differences in IFN-α and β induction between the GI and GIII strains. A series of chimeric recombinant viruses were generated by exchanging viral proteins between SH7 and SH15 strains on the rGI and rGIII backbones (Fig 3A). The generated chimeric recombinant viruses were tested for changes in induction of IFN-α and β production at the mRNA level in DEF. We first determined whether the structural proteins played roles in the differences in IFN-α and β induction between the strains. The chimeric virus rGI/SH15CPrME was generated by substitution of SH15 structural proteins on the rGI backbone, whereas rGIII/SH7CPrME was constructed by substitution of the SH7 structural proteins on the rGIII backbone (Fig 3A). DEF were infected with the chimeric viruses with exchange of structural proteins (C, PrM, and E) and the respective parental recombinant viruses (rGI and rGIII) at a multiplicity of infection (MOI) of 1. As shown in S4A Fig, rGI/SH15CPrME and rGIII/SH7CPrME induced levels of IFN-α and β expression similar to those induced by the parental rGI and rGIII, respectively, thus suggesting that the structural proteins had no significant role in determining the differences in IFN-α and β induction between the GI and GIII strains.

To determine whether the non-structural proteins played essential roles in determining the differences in IFN-α and β induction between the GI and GIII strains, we generated a series of chimeric recombinant viruses with exchange of non-structural proteins (NS1, NS2A, NS2B/NS3, NS4A/NS4B, and NS5) (Fig 3A) and tested for the changes in induction of IFN-α and β expression at the mRNA level in DEF. All chimeric viruses excluding rGI/SH15NS5 and rGIII/SH7NS5 induced levels of IFN-α and β expression similar to those induced by the parental recombinant viruses (Figs 3A and S4B), thus suggesting that only NS5 had a significant role in determining the differences in IFN-α and β induction between the strains.

rGI/SH15NS5 with the substitution of SH15 NS5 on the rGI backbone induced significantly higher levels of IFN-α and β expression than the parental rGI. In contrast, rGIII/SH7NS5 with the substitution of SH7 NS5 on the rGIII backbone resulted in lower levels of IFN-α and β expression than the parental rGIII (Figs 3A and S4B). These results suggested that NS5 played an essential role in determining the differences in IFN-α and β induction between the strains. To confirm these results, we further infected DEF with rGI/SH15NS5 and rGIII/SH7NS5 at different MOI (0.5, 1 and 5) and examined changes in induction of IFN-α and β expression at different time points. Substitution of NS5 with other sequences altered the ability of the parental virus to induce IFN-α and β expression at both the mRNA (Fig 3B and 3C) and protein levels (Fig 3D and 3E), thus confirming that NS5 played an essential role in determining the differences in IFN-α and β induction between the GI and GIII strains.

**Fig 4:**
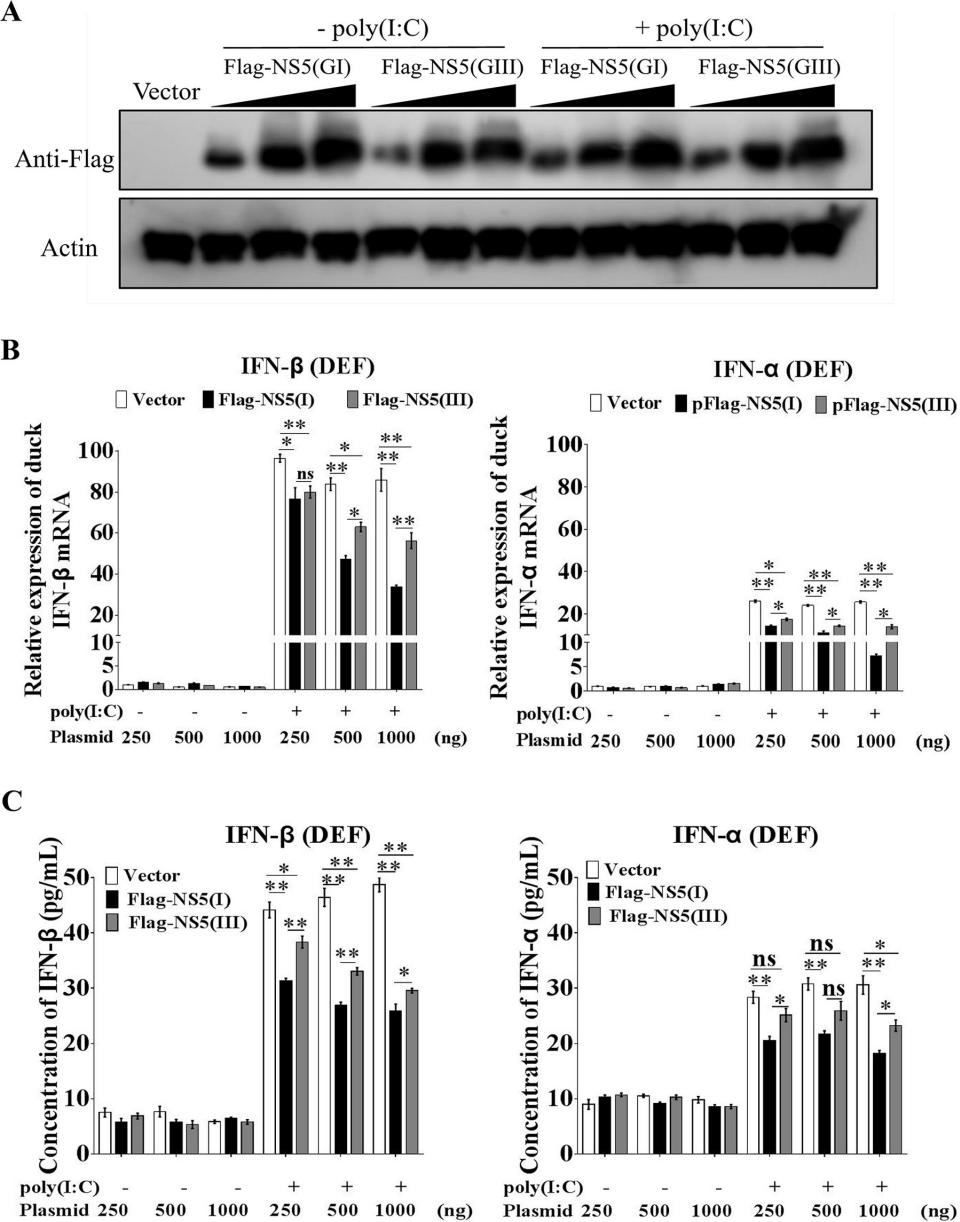
Inhibitory effects of NS5 on IFN-α and β expression. DEF were transfected with plasmids for expression of Flag-NS5(GI) or Flag-NS5(GIII) at amounts of 250, 500, and 1,000 ng and incubated for 24 h. The transfectants were mock-treated (-poly(I:C)) or treated with poly(I:C) (+poly(I:C)) for 12 h. (A) The expression of Flag-NS5(GI) and Flag-NS5(GIII) in the transfectants was detected by western blotting with anti-Flag antibodies. (B) The mRNA levels of IFN-α and β in the cell pellets were determined by using qRT-PCR. (C) The concentrations of IFN-α and β proteins in the supernatants were determined by using ELISA. All data are presented as mean ± SD from three independent experiments. **, *p* < 0.01; *, *p* < 0.05; ns, no significant difference, by Student’s *t*-test.

To provide further evidence of the role of NS5 in determining the differences in IFN-α and β production between the GI and GIII strains, we tagged NS5 from SH7 (Flag-NS5(GI)) and SH15 (Flag-NS5(GIII)) with Flag and expressed them in DEF to examine the effects on IFN-α and β expression induced by poly(I:C) stimulation[36]. The exogenous expression of Flag-NS5(GI) and Flag-NS5(GIII) in the transfectants was confirmed by western blotting with anti-Flag antibodies (Fig 4A). Expression of both Flag-NS5(GI) and Flag-NS5(GIII) inhibited the production of IFN-α and β induced by poly(I:C) stimulation at both the mRNA (Fig 4B) and protein levels (Fig 4C) in a dose-dependent manner. However, comparison of the inhibitory effects between Flag-NS5(GI) and Flag-NS5(GIII) indicated that the levels of IFN-α and β expression in cells expressing Flag-NS5(GI) were markedly lower than those in cells expressing Flag-NS5(GIII) at both the mRNA (Fig 4B) and protein (Fig 4C) levels in the presence of poly(I:C) stimulation, thus demonstrating that the inhibitory effects of NS5 on IFN-α and β production differed between the strains: NS5 inhibited IFN-α and β expression more efficiently in of GI strains than GIII strains, thereby leading to lower levels of IFN-α and β production in GI-infected cells than GIII-infected cells. Overall, these observations demonstrated that NS5 was the viral determinant of the differences in IFN-α and β induction between the GI and GIII strains.

### NS5 determines the differences in replication efficiency between the GI and GIII strains in DEF

**Fig 5:**
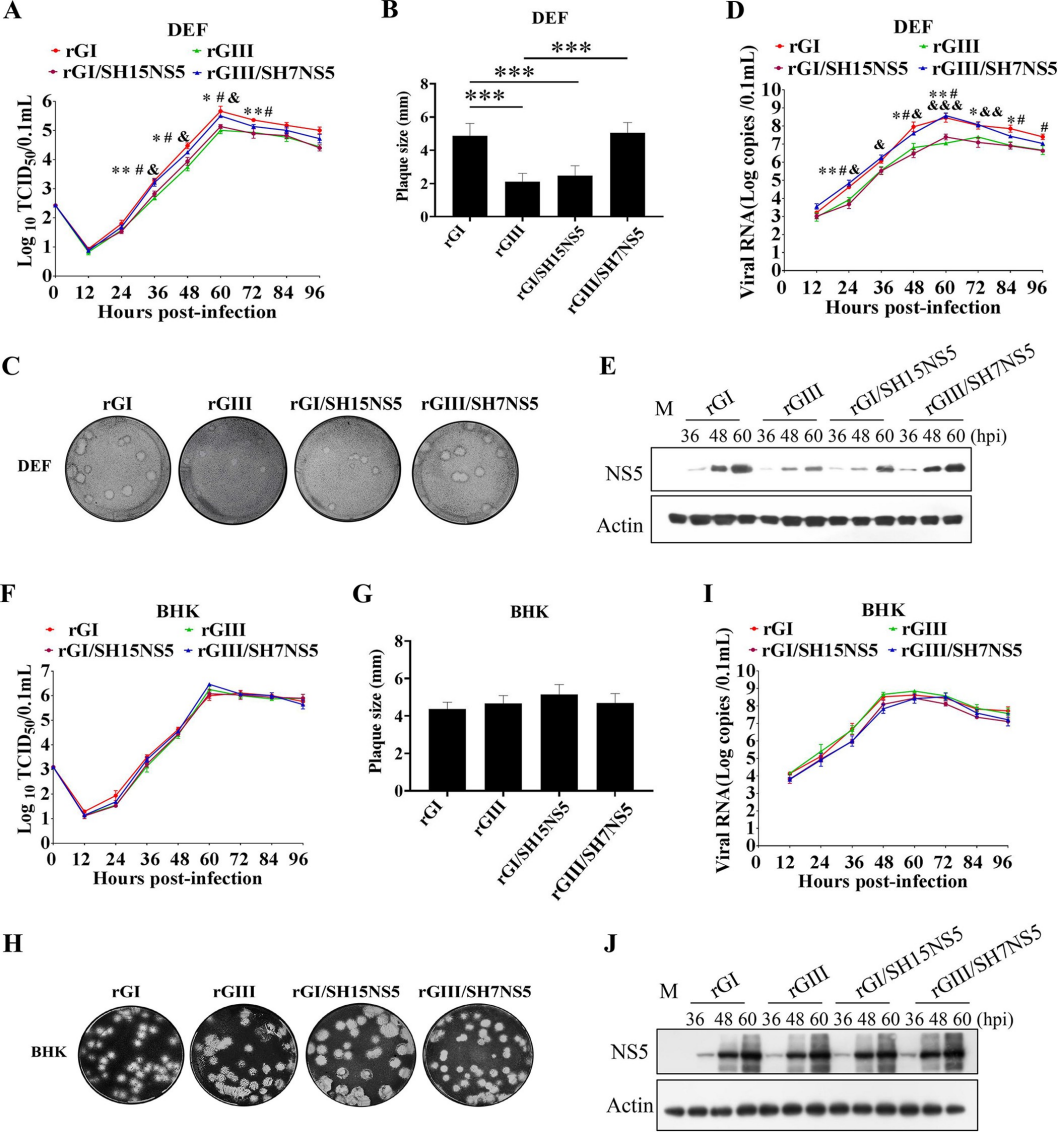
Replication efficiency of chimeric viruses with substitution of NS5. (A, D and E) DEF were infected with the indicated chimeric viruses and the respective parental viruses at 0.01 MOI and collected at the indicated time points for analysis of replication efficiency. The replication titers in the supernatants were titrated with TCID_50_ assays in BHK cells (A). The viral RNA copies and NS5 levels in the cells were examined with TaqMan probe-based qRT-PCR (D) and western blotting with anti-NS5 antibodies (E), respectively. All data are presented as mean ± SD from three independent experiments and were tested by Student’s *t*-test. The significant differences between rGI and rGIII at different time points are labeled (*, *p* < 0.05; **, *p* < 0.01). The significant difference between rGI and rGI/SH15NS5 at different time points is marked (#, *p* < 0.05). The significant difference between rGIII and rGIII/SH7NS5 at different time points is indicated (&&&, *p* < 0.001; &&, *p* < 0.01; &, *p* < 0.05). (B, C, G and H) Monolayers of DEF (B and C) or BHK cells (G and H) were infected with the indicated chimeric viruses and the respective parental viruses at 100 PFU for analysis of plaque morphology. The plaques were stained with crystal violet at 4 dpi (C and H) and the plaque diameters were measured and plotted (B and G). The significant differences between groups were tested by Student’s *t*-test (***, *p* < 0.001). (F, I and J) BHK cells were infected with the indicated chimeric viruses and the respective parental viruses at 0.01 MOI and collected at the indicated time points for analysis of replication efficiency. The replication titers in the supernatants were titrated with TCID_50_ assays in BHK cells (F). The viral RNA copies and NS5 levels in the cells were examined with TaqMan probe-based qRT-PCR (I) and western blotting with anti-NS5 antibodies (J), respectively.

Because NS5 was found to be the viral determinant of the differences in IFN-α and β induction between the GI and GIII strains, we examined whether NS5 might determine the differences in replication efficiency between the strains. DEF were infected with the chimeric recombinant viruses with exchange of structural proteins or non-structural proteins (Figs 5A and S4C) as well as parental rGI and rGIII, and the replication kinetics was monitored with TCID_50_ assays. All the recombinant viruses reached peak titers at 60 hpi (Figs 5A and S4C). The viral titers of rGI were higher than those of rGIII, and a significant difference was observed from 36 to 72 hpi (Fig 5A), in agreement with the observations in Figs 1K and 2A. Analysis of plaque sizes formed in DEF indicated that rGI formed large plaques approximately 2-fold larger than those formed by rGIII (Fig 5B and 5C). Similar differences in viral titers (S4C Fig) and plaque sizes (S4D and S4E Fig) were observed within the chimeric virus pair of rGI/SH15CPrME and rGIII/SH7CPrME, rGI/SH15NS1 and rGIII/SH7NS1, rGI/SH15NS2A and rGIII/SH7NS2A, rGI/SH15NS2B/NS3 and rGIII/SH7NS2B/NS3, and rGI/SH15NS4A/NS4B and rGIII/SH7NS4A/NS4B, suggesting that the structural proteins (C, PrM, and E), NS1, NS2A, NS2B/NS3, and NS4A/NS4B had no significant role in determining the differences in replication efficiency between the GI and GIII strains.

However, the viral titers of rGI/SH15NS5 were clearly lower than those of the parental rGI but similar to those of rGIII. Additionally, rGIII/SH7NS5 showed replication titers 0.5–0.7 log higher than those of the parental rGIII from 36 to 60 hpi, values close to the viral titers of rGI (Fig 5A). Analysis of plaque sizes formed in DEF indicated the plaque size of rGI/SH15NS5 was smaller than that of the parental rGI, similarly to the results for rGIII, whereas the plaque size of rGIII/SH7NS5 was larger than that of the parental rGIII, similarly to the results for rGI (Fig 5B and 5C). Since RNA replication is requisite for the IFN-I response to flaviviruses, we measured the viral RNA copies in the infected DEF by a TaqMan probe-based qRT-PCR. The viral RNA copies in DEF infected with rGI/SH15NS5 were significantly lower than those of the parental rGI but similar to those of rGIII. While the viral RNA copies in DEF infected with rGIII/SH7NS5 were clearly higher than those of the parental rGIII but similar to those of rGI (Fig 5D). These changes in viral RNA copies were coincident with the levels of IFN-α and β production observed in Fig 3C and 3E. In addition, the levels of viral NS5 protein in DEF infected with rGI and rGIII/SH7NS5 were notably higher than those in DEF infected with rGI/SH15NS5 and rGIII, respectively, at 48 and 60 hpi (Fig 5E). These findings indicated that NS5 determined the differences in replication efficiency between the GI and GIII strains in DEF, thus suggesting that the inhibitory effects of NS5 on IFN-α and β expression corelated positively with the replication efficiency. In addition, NS5 from GI and GIII strains showed no significant difference in inhibition of GI and GIII replication in baby hamster kidney (BHK) cells. The replication titers (Fig 5F), plaque sizes (Fig 5G and 5H), viral RNA copies (Fig 5I), and the levels of NS5 protein (Fig 5J) were similar among all recombinant viruses in BHK cells.

### A region harboring two amino acid variations at position 372 and 386 of NS5 is responsible for the differences in IFN-α and β induction and replication efficiency between the GI and GIII strains in DEF

**Fig 6:**
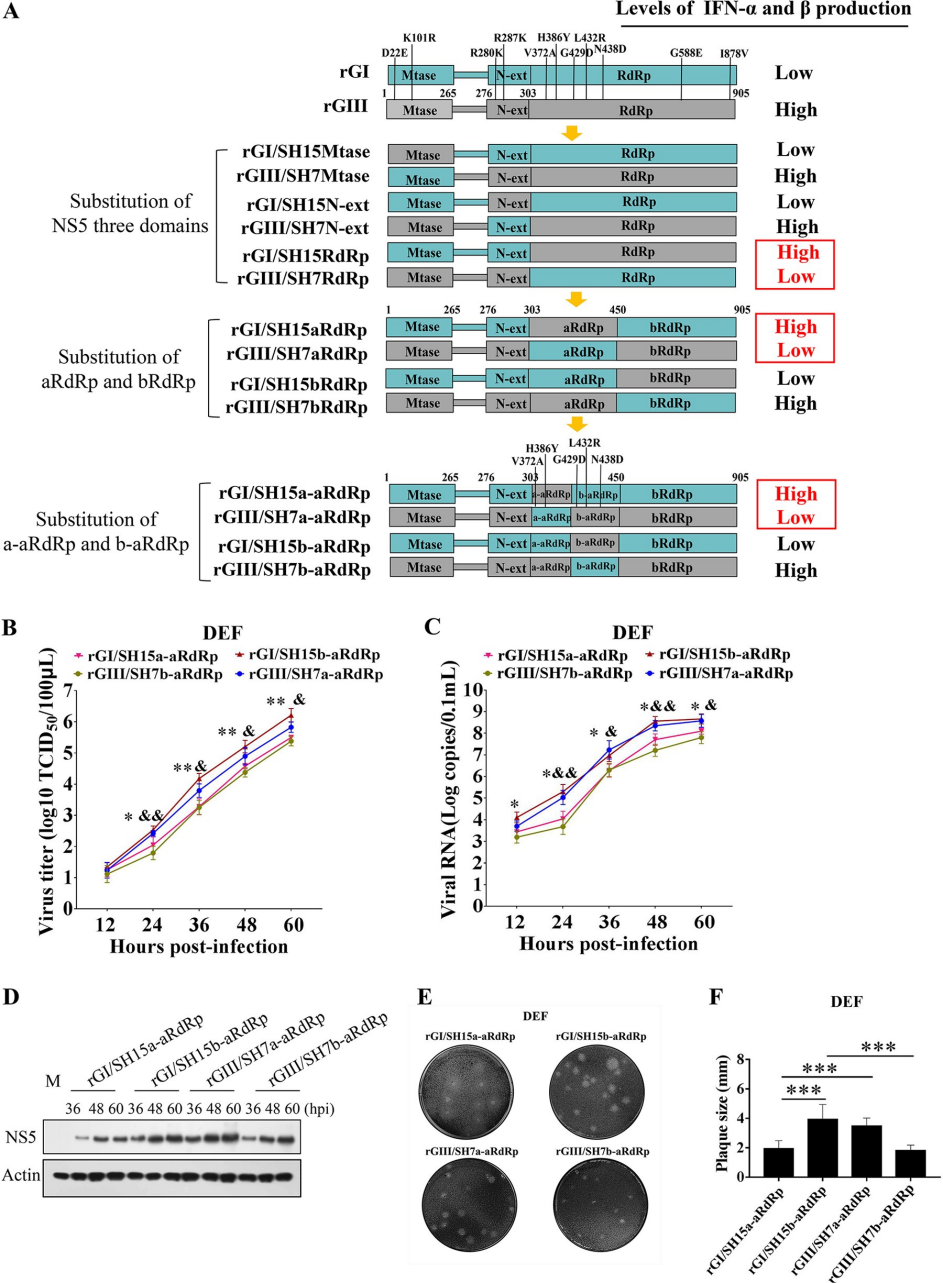
Identification of the region of NS5 responsible for the differences in IFN-α and β induction and replication efficiency. (A) Schematic diagram of chimeric recombinant viruses used in this study. Viral proteins of GI and GIII strains are highlighted in blue and gray, respectively. (B, C and D) DEF were infected with the indicated chimeric viruses at 0.01 MOI and collected at the indicated time points for analysis of replication efficiency. The replication titers in the supernatants were titrated with TCID_50_ assays in BHK cells (B). The viral RNA copies and NS5 levels in the cells were examined with TaqMan probe-based qRT-PCR (C) and western blotting with anti-NS5 antibodies (D), respectively. All data are presented as mean ± SD from three independent experiments and were tested by Student’s *t*-test. The significant difference between rGI/SH15a-aRdRp and rGI/SH15b-aRdRp at different time points is marked (*, *p* < 0.05; **, *p* <0.01). The significant difference between rGIII/SH7a-aRdRp and rGIII/SH7b-aRdRp at different time points is labeled (&, *p* < 0.05; &&, *p* < 0.01). (E and F) Monolayers of DEF were infected with the indicated chimeric viruses at 100 PFU for analysis of plaque morphology. The plaques were stained with crystal violet at 4 dpi (E) and the plaque diameters were measured and plotted (F). The significant differences between groups were tested by Student’s *t*-test (***, *p* < 0.001).

A total of 11 amino acid variations between the GI and GIII strains were distributed in NS5 (Fig 6A and Table 1), the largest viral protein consisting of an N-terminal methyltransferase (MTase), an N-terminal extension (N-ext), and a C-terminal RNA-dependent RNA polymerase (RdRp) domain[37]. To identify the amino acid variation(s) responsible for the differences in IFN-α and β induction between the GI and GIII strains, we generated a series of chimeric recombinant viruses (rGI/SH15MTase, rGI/SH15N-ext, rGI/SH15RdRp, rGIII/SH7MTase, rGIII/SH7N-ext, and rGIII/SH7RdRp) with exchange of the three domains between the GI and GIII strains (Fig 6A), which were tested for changes in IFN-α and β induction in DEF. All chimeric recombinant viruses excluding rGI/SH15RdRp and rGIII/SH7RdRp induced similar levels of IFN-α and β expression to those induced by the parental viruses (Figs 6A and S5A), thus suggesting that RdRp, but not MTase and N-ext, had a significant role in determining the differences in IFN-α and β induction between the strains.

Chimeric rGI/SH15RdRp with a substitution of SH15 RdRp on the rGI backbone induced higher levels of IFN-α and β expression than those induced by the parental rGI. In contrast, the chimeric rGIII/SH7RdRp with the substitution of SH7 RdRp on the rGIII backbone showed lower levels of IFN-α and β expression than those induced by the parental rGIII (Figs 6A and S5A), thus suggesting that the RdRp domain was responsible for the differences in IFN-α and β induction between the strains.

There were seven amino acid variations present in the RdRp domain between the GI and GIII strains (Fig 6A). To narrow the region of the RdRp domain responsible for the differences in IFN-α and β induction, we further divided the RdRp domain into two parts: aRdRp, containing five amino acid variations, and bRdRp, containing two amino acid variations. We then generated four chimeric recombinant viruses (rGI/SH15aRdRp, rGI/SH15bRdRp, rGIII/SH7aRdRp, and rGIII/SH7bRdRp) by exchange of aRdRp and bRdRp between the GI and GIII strains (Fig 6A). The effects of these chimeric viruses on IFN-α and β induction were tested in DEF. As shown in Figs 6A and S5B, the chimeric rGI/SH15aRdRp and rGIII/SH7aRdRp induced different levels of IFN-α and β expression from those induced by parental rGI and rGIII, respectively, thus suggesting that the aRdRp region was responsible for the differences in IFN-α and β induction between the strains.

The aRdRp region was further divided into two parts: a-aRdRp, containing two amino acid variations, and b-aRdRp, including three amino acid variations. These were then used to generate four chimeric recombinant viruses (rGI/SH15a-aRdRp, rGI/SH15b-aRdRp, rGIII/SH7a-aRdRp, and rGIII/SH7b-aRdRp) by exchange of a-aRdRp and b-aRdRp between the GI and GIII strains (Fig 6A). The effects of these chimeric viruses on IFN-α and β induction were tested in DEF. As shown in Figs 6A and S5C, the levels of IFN-α and β expression induced by rGI/SH15a-aRdRp and rGIII/SH7a-aRdRp significantly differed from those induced by parental rGI and rGIII, respectively, thus indicating that the a-aRdRp region containing two amino acid variations at positions 372 and 386 was responsible for the differences in IFN-α and β induction between the strains.

To test whether the differences in IFN-α and β induction resulted in the differences in replication efficiency, we examined the replication titers of chimeric viruses with exchange of NS5 domains or aRdRp and bRdRp between the GI and GIII strains (Fig 6A) in DEF. All chimeric viruses excluding the chimeric virus rGI/SH15RdRp, rGIII/SH7RdRp, rGI/SH15aRdRp and rGIII/SH7aRdRp showed replication titers (S5D Fig) and plaque sizes (S5E Fig) similar to those of the parental recombinant viruses, thus suggesting that NS5 aRdRp region that was responsible for the differences in IFN-α and β induction had a significant role in determining the differences in replication efficiency between the strains.

Analysis of replication titers of the chimeric virus rGI/SH15a-aRdRp, rGI/SH15b-aRdRp, rGIII/SH7a-aRdRp, and rGIII/SH7b-aRdRp in DEF reveled that the viral titers of rGI/SH15b-aRdRp and rGIII/SH7a-aRdRp containing the a-aRdRp region of GI strains were markedly higher than those of rGI/SH15a-aRdRp and rGIII/SH7b-aRdRp harboring the a-aRdRp region of GIII strains (Fig 6B). Additionally, the viral RNA copies (Fig 6C) and NS5 levels (Fig 6D) of rGI/SH15b-aRdRp and rGIII/SH7a-aRdRp were noticeably higher than those of rGI/SH15a-aRdRp and rGIII/SH7b-aRdRp. Analysis of plaque sizes showed that the plaque sizes of rGI/SH15b-aRdRp and rGIII/SH7a-aRdRp were clearly larger than those of rGI/SH15a-aRdRp and rGIII/SH7b-aRdRp (Fig 6E and 6F). These differences were not observed in BHK cells, and the replication titers (S6A Fig) and plaque sizes (S6B Fig) were similar among the chimeric recombinant virus rGI/SH15b-aRdRp, rGIII/SH7a-aRdRp, rGI/SH15a-aRdRp and rGIII/SH7b-aRdRp. Together, these data indicated that the a-aRdRp region harboring two amino acid variations at position 372 and 386 of NS5 was responsible for the differences in IFN-α and β induction, thereby resulting in the differences in replication efficiency between the strains in DEF.

### Combination of NS5-V372A and NS5-H386Y variations is essential for GI strains to induce low levels of IFN-α and β expression

**Fig 7:**
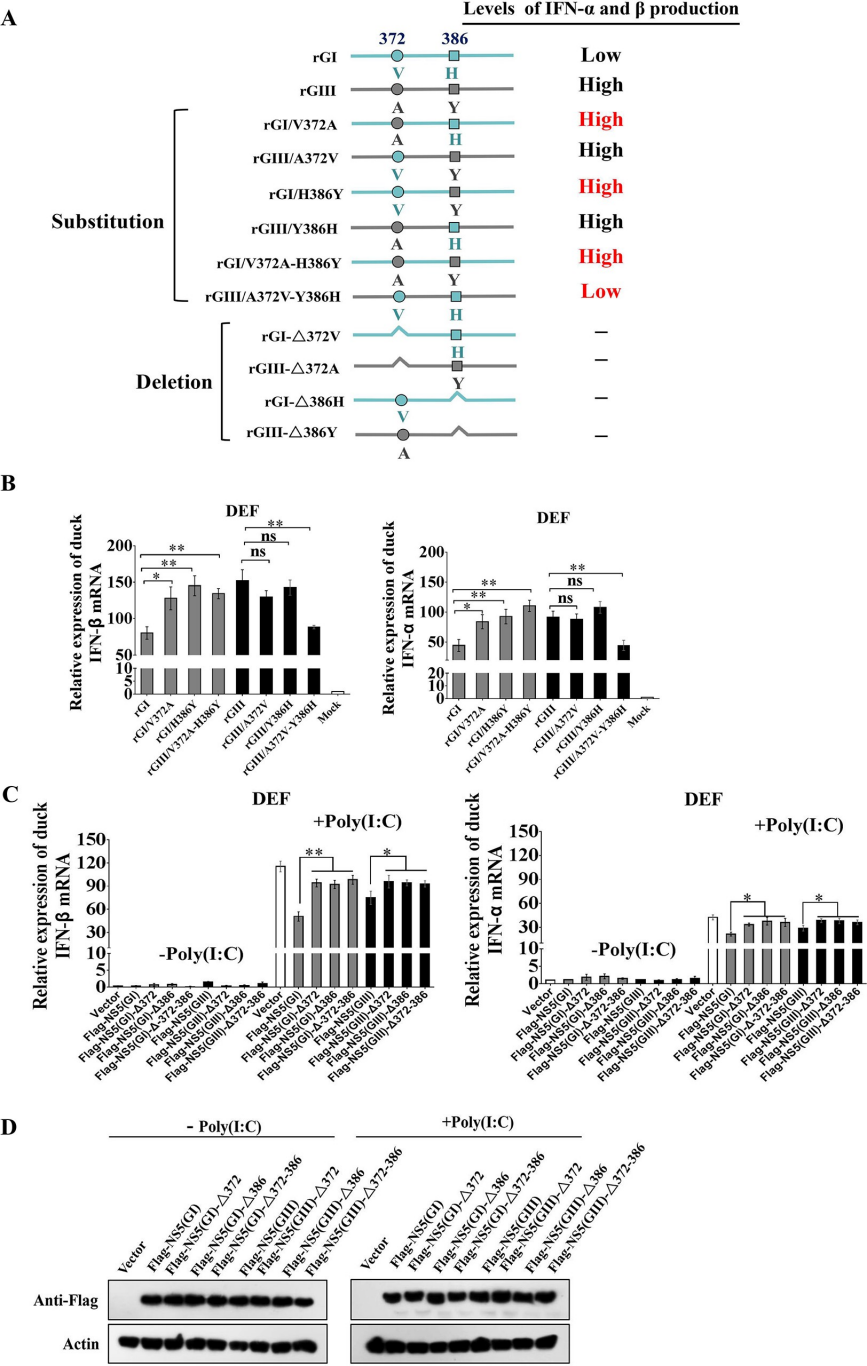
Effects of NS5-V372A and NS5-H386Y variations on IFN-α and β induction. (A) Schematic diagram of substitution mutant viruses used in this study. Viral proteins of GI and GIII strains are highlighted in blue and gray, respectively. (B) DEF were infected with the indicated substitution mutant viruses at 1 MOI and harvested at 24 hpi for measurement of IFN-α and β production at the mRNA level by qRT-PCR. (C and D) DEF were transfected with the indicated plasmids and incubated for 24 h. The transfectants were mock-treated (-poly(I:C)) or treated with poly(I:C) (+poly(I:C)) for 12 h. The mRNA levels of IFN-α and β in the cell pellets were determined by using qRT-PCR (C). The expression of Flag-NS5 and Flag-NS5 deletion mutants were analyzed with western blotting with anti-Flag antibodies (D). All data are presented as mean ± SD from three independent experiments. **, *p* < 0.01; *, *p* < 0.05; ns, no significant difference, by Student’s *t*-test.

Given that the a-aRdRp region of NS5 was found to be responsible for the differences in IFN-α and β induction between the GI and GIII strains, which harbored two amino acid variations at positions 372 (NS5-372) and 386 (NS5-386), we therefore analyzed the conservation of amino acid residues at NS5-372 and NS5-386 in 71 GI and in 99 GIII strains. A valine (Val, V) at NS5-372 and a histidine (His, H) at NS5-386 were well conserved in all GI strains, which aligned with a conservation rate of 100% (71/71) (Table 2). However, these residues in GIII strains were relatively nonconserved: 90.9% (90/99) had an alanine (Ala, A) and 9.1% (9/99) had a Val at NS5-372; and 46.5% (46/99) had a His and 53.5% (53/99) had a tyrosine (Tyr, Y) at NS5-386 (Table 2). To test the contribution of each variant residue to the differences in IFN-α and β induction between the GI and GIII strains, we generated three substitution mutant viruses, rGI/V372A (Val to Ala substitution at NS5-372), rGI/H386Y (His to Tyr substitution at NS5-386), and rGI/V372A-H386Y (Val to Ala substitution at NS5-372 plus His to Tyr substitution at NS5-386) based on the rGI backbone, and generated three substitution mutant viruses, rGIII/A372V (Ala to Val substitution at NS5-372), rGIII/Y386H (Tyr to His substitution at NS5-386), and rGIII/A372V-Y386H (Ala to Val substitution at NS5-372 plus Tyr to His substitution at NS5-386) based on the rGIII backbone (Fig 7A). In addition, we were unable to rescue a recombinant virus with a single deletion of residue either at NS5-372 or NS5-386, thus suggesting that these two residues were critical for JEV replication.

**Table 2:** Conservation of residues at NS5-372 and NS5-386 in GI and in GIII strains.

		Conservation rate (%)			
		NS5-372		NS5-386	
**Genotype**	**Number of strains**	V (Val)	A (Ala)	H (His)	Y (Tyr)
**GI**	71	100% (71/71)	0	100% (71/71)	0
**GIII**	99	9.1% (9/99)	90.9% (90/99)	46.5% (46/99)	53.5% (53/99)

These six substitution mutant viruses were tested for changes in IFN-α and β induction in DEF. The levels of IFN-α and β expression induced by rGI/V372A, rGI/H386Y, or rGI/V372A-H386Y were significantly higher than those induced by the parental rGI (Fig 7A and 7B), thus suggesting that either a Val to Ala substitution at NS5-372 or a His to Tyr substitution at NS5-386 impaired the ability of GI strains to induce low levels of IFN-α and β expression. Additionally, the levels of IFN-α and β expression induced by rGIII/A372V and rGIII/Y386H were similar to those induced by the parental rGIII, whereas the levels of IFN-α and β expression induced by rGIII/A372V-Y386H were significantly lower than those induced by the parental rGIII (Fig 7A and 7B). Furthermore, no significant differences in IFN-α and β induction among rGI, rGIII, rGI/V372A-H386Y and rGIII/A372A-Y386H were observed in DEF, in which IFN-α and β expression was silenced by siRNA (Fig 2D and 2E), The effects of NS5-V372A and NS5-H386Y variations on IFN-α and β expression were further tested in ST and bEnd.3 cells, no significant differences in IFN-α and β expression were observed among the cells infected with rGI, rGIII, rGI/V372A-H386Y and rGIII/A372A-Y386H (S7A and S7B Fig). Together, these data suggested that the combination of NS5-V372A and NS5-H386Y variations was essential for GI strains to induce the low levels of IFN-α and β expression in DEF.

To further examine the roles of NS5-372 and NS5-386 in IFN-α and β induction, we generated a series of plasmids for expression of Flag-NS5(GI)-Δ372 (GI NS5 with NS5-372 deletion), Flag-NS5(GI)-Δ386 (GI NS5 with NS5-386 deletion), Flag-NS5(GI)-Δ372–386 (GI NS5 with double deletions of NS5-372 and NS5-386), Flag-NS5(GIII)-Δ372 (GIII NS5 with NS5-372 deletion), Flag-NS5(GIII)-Δ386 (GIII NS5 with NS5-386 deletion), and Flag-NS5(GIII)-Δ372–386 (GIII NS5 with double deletions of NS5-372 and NS5-386). We then tested the plasmids for inhibition of IFN-α and β expression induced by poly(I:C) stimulation in DEF. In the presence of poly(I:C) stimulation, the levels of IFN-α and β expression in all cells expressing the NS5 deletion mutants were significantly higher than those in the cells expressing wild type NS5 (Flag-NS5(GI) or Flag-NS5(GIII)) (Fig 7C), thus suggesting that NS5-372 and NS5-386 were essential for NS5 to inhibit IFN-α and β expression. The expression of Flag-NS5 and Flag-NS5 deletion mutants in the transfectants was confirmed by western blotting with anti-Flag antibodies (Fig 7D).

### Combination of NS5-V372A and NS5-H386Y variations is essential for GI strains to replicate more efficiently than GIII strains in DEF

**Fig 8:**
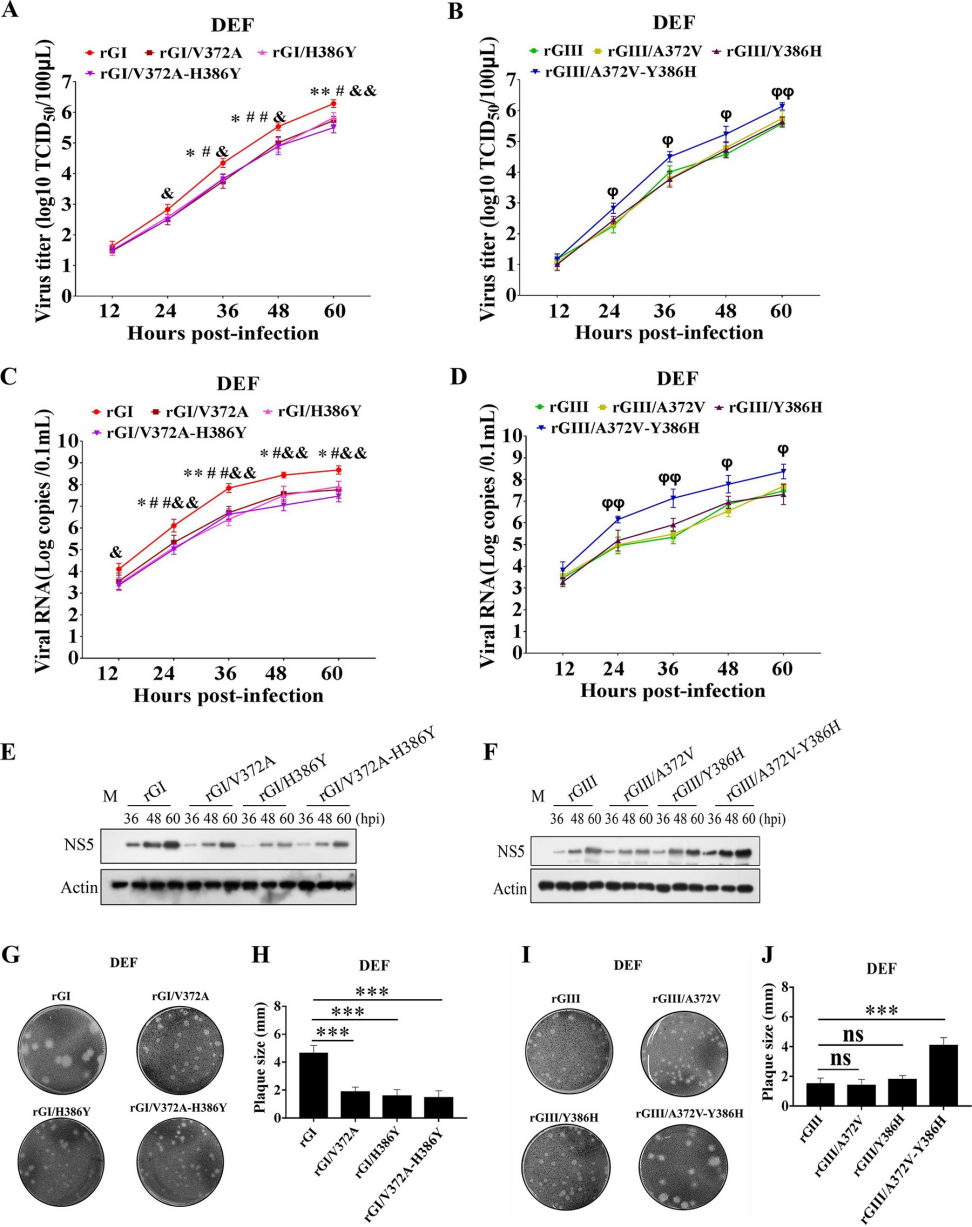
Effects of NS5-V372A and NS5-H386Y variations on viral replication. (A to F) DEF were infected with the indicated substitution mutant viruses at 0.01 MOI and collected at the indicated time points for analysis of replication efficiency. The replication titers in the supernatants were titrated with TCID_50_ assays in BHK cells (A and B). The viral RNA copies and NS5 protein levels in the cells were examined with TaqMan probe-based qRT-PCR (C and D) and western blotting with anti-NS5 antibodies (E and F), respectively. All data are presented as mean ± SD from three independent experiments and were tested by Student’s *t*-test. The significant difference between rGI and rGI/V372A is labeled (**, *p* < 0.01; *, *p* < 0.05). The significant difference between rGI and rGI/H386Y is marked (##, *p* < 0.01; #, *p* < 0.05). The significant difference between rGI and rGI/V372A-H386Y is indicated (&&, *p* < 0.01; &, *p* < 0.05). The significant difference between rGIII and rGIII/A372V-Y386H is indicated (φφ, *p* < 0.01; φ, *p* < 0.05). (G to J) Monolayers of DEF were infected with the indicated substitution viruses at 100 PFU for analysis of plaque morphology. The plaques were stained with crystal violet at 4 dpi (G and I) and the plaque diameters were measured and plotted (H and J). The significant differences between groups were tested by Student’s *t*-test (***, *p* < 0.001).

Given that the NS5-V372A and NS5-H386Y variations were found to be essential for GI strains to induce low levels of IFN-α and β expression, we examined whether NS5-V372A and NS5-H386Y variations were essential for GI strains to replicate more efficiently than GIII strains. DEF were infected with the substitution mutant rGI/V372A, rGI/H386Y, and rGI/V372A-H386Y based on the rGI backbone, and the substitution mutant rGIII/A372V, rGIII/Y386H and rGIII/A372V-Y386H based on the rGIII backbone, and then the growth kinetics and viral RNA copies were monitored with TCID_50_ assays (Fig 8A and 8B) and TaqMan probe-based qRT-PCR (Fig 8C and 8D), respectively. rGI/V372A, rGI/H386Y, and rGI/V372A-H386Y yielded viral titers 0.45–0.8 log lower than those of the parental rGI from 36 to 60 hpi (Fig 8A). Additionally, the viral titers of rGIII/A372V and rGIII/Y386H were similar to those of rGIII, but rGIII/A372V-Y386H produced viral titers markedly higher than those of rGIII, rGIII/A372V, and rGIII/Y386H from 24 to 60 hpi (Fig 8B). The viral RNA copies of rGI/V372A, rGI/H386Y, and rGI/V372A-H386Y were lower than those of rGI from 24 to 60 hpi (Fig 8C), while the viral RNA copies of rGIII/A372V and rGIII/Y386H were similar to those of rGIII, but rGIII/A372V-Y386H showed viral RNA copies significantly higher than those of rGIII from 24 to 60 hpi (Fig 8D). Analysis of the protein levels of NS5 in the infected DEF revealed that the levels of NS5 in DEF infected with rGI/V372A, rGI/H386Y, and rGI/V372A-H386Y were noticeably lower than those in DEF infected with rGI (Fig 8E), while the levels of NS5 in DEF infected with rGIII/A372V-Y386H was higher than those in DEF infected with rGIII, rGIII/A372V, and rGIII/Y386H (Fig 8F). The plaques formed by rGI/V372A, rGI/H386Y, and rGI/V372A-H386Y were clearly smaller than those formed by the parental rGI (Fig 8G and 8H). In contrast, rGIII/A372V-Y386H formed larger plaques than those formed by rGIII, rGIII/A372V, and rGIII/Y386H (Fig 8I and 8J). These data demonstrated that the combination of NS5-V372A and NS5-H386Y variations contributed to the high levels of GI replication in DEF. Furthermore, no significant differences in replication titers among rGI, rGIII, rGI/V372A-H386Y, and rGIII/A372A-Y386H were observed in DEF, in which IFN-α and β expression was silenced by siRNA (Fig 2F), suggesting that the contribution of NS5-V372A and NS5-H386Y variations to the high levels of GI replication was dependent on IFN-α and β in DEF.

These changes in replication efficiency were not observed in BHK, ST, bEnd.3, and C6/36 cells. The replication titers (S6C Fig) and plaque sizes (S6D Fig) in BHK cells were similar among the six substitution mutant viruses. The replication titers and NS5 protein levels in ST (S7C and S7D Fig), bEnd.3 (S7E and S7F Fig), and C6/36 cells (S7G and S7H Fig) were no significant differences among rGI, rGI/V372A-H386Y, rGIII, and rGIII-A372V-Y386H.

### The number of hydrogen bonds formed by NS5-372 and NS5-386 contributes to the differences in IFN-α and β induction and replication efficiency between the GI and GIII strains in DEF

**Fig 9:**
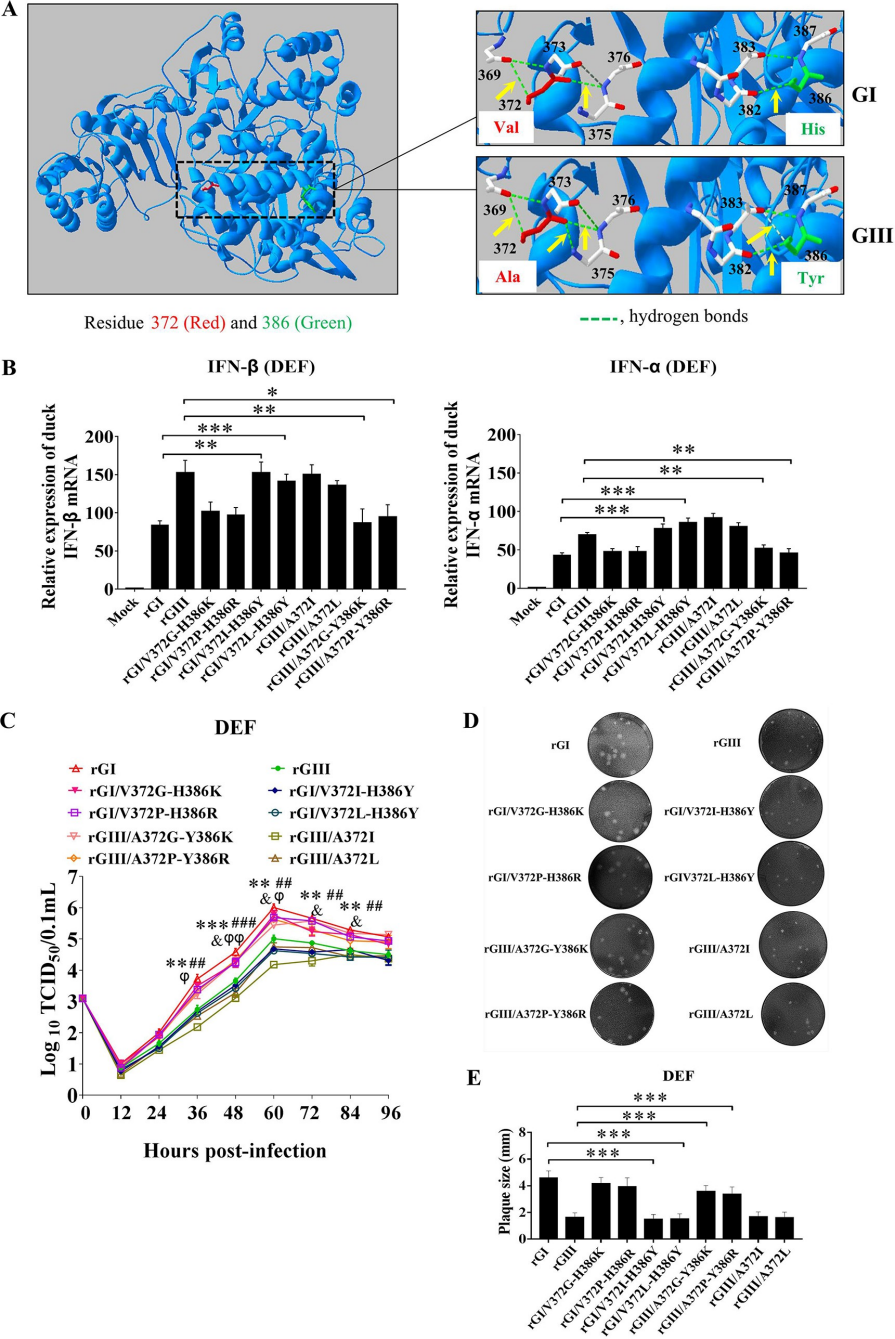
Effects of the number of hydrogen bonds on IFN-α and β expression and viral replication. (A) 3D structure of NS5. NS5-372 and NS5-386 are highlighted in red and green, respectively. Green broken lines represent hydrogen bonds. The hydrogen bonds formed by NS5-372 and NS5-386 with their neighboring residues are indicated by yellow arrows. (B) DEF were infected with the indicated substitution mutant viruses at 1 MOI and harvested at 24 hpi for measurement of IFN-α and β production at the mRNA level by qRT-PCR. *, *p* < 0.05; **, *p* < 0.01; ***, *p* < 0.001, by Student’s *t*-test. (C) DEF were infected with the indicated substitution mutant viruses at 0.01 MOI for analysis of replication efficiency. The supernatants were sampled at the indicated time points and titrated with TCID_50_ assays on BHK cells. Significant difference between rGI and rGI/V372I-H386Y is labeled (***, *p* < 0.001; **, *p* < 0.01). Significant difference between rGI and rGI/V372L-H386Y is marked (###, *p* < 0.001; ##, *p* < 0.01). Significant difference between rGIII and rGIII/A372G-Y386K is indicated (&, *p* < 0.05). Significant difference between rGIII and rGIII/A372P-Y386R is indicated (φφ, *p* < 0.01; φ, *p* < 0.05). (D and E) Monolayers of DEF were infected with the indicated substitution viruses at 100 PFU for analysis of plaque morphology. The plaques were stained with crystal violet at 4 dpi (D) and the plaque diameters were measured and plotted €. The significant differences between groups were tested by Student’s *t*-test (***, *p* < 0.001).

Structurally, NS5-372 and NS5-386 were located in the α-helix of the nuclear localization signal (NLS) region, which is involved in interaction with importins, antagonism of IFN-I production, and viral replication[36, 38]. Analysis of the three-dimensional structure of NS5 revealed a difference in the number of hydrogen bonds formed by NS5-372 and NS5-386 with their neighboring residues between the GI and GIII strains. NS5-372 of the GI strains formed one hydrogen bond with residues 369 and 375, whereas NS5-386 formed one hydrogen bond with residue 382. Interestingly, NS5-372 of the GIII strains formed one hydrogen bond with residues 369, 375, and 376, whereas NS5-386 formed one hydrogen bond with residues 382 and 383 (Fig 9A, yellow arrow indicates the hydrogen bond). This analysis indicated that NS5-372 and NS5-386 of GI strains formed fewer hydrogen bonds with their neighboring residues than GIII strains, thus potentially resulting in a higher flexibility of the NS5 NLS region in GI relative to GIII.

The flexibility of flavivirus NS5 allows it to adopt distinct conformations and has an important effect on the diverse functions of NS5[39, 40]. To explore whether the number of hydrogen bonds formed by NS5-372 and NS5-386 contributed to the differences in IFN-α and β induction and replication efficiency between the GI and GIII strains, we first analyzed the number of hydrogen bonds formed by different amino acids at NS5-372 and NS5-386 (S2 Table). On the basis of the number of hydrogen bonds and the properties of amino acids, Gly, Pro, Ile, and Leu belong to the same nonpolar group as Val and Ala and were therefore selected for replacement of Val or Ala at NS5-372, and Lys and Arg from the same polar basic group including His were selected for replacement of His or Tyr at NS5-386 (Table 3). Four substitution mutants, rGI/V372G-H386K, rGI/V372P-H386R, rGI/V372I-H386Y, and rGI/V372L-H386Y, were generated on the rGI backbone by replacement of Val with Gly, Pro, Ile, or Leu at NS5-372, and of His with Lys, Arg, or Tyr at NS5-386. Four substitution mutants, rGIII/A372I, rGIII/A372L, rGIII/A372G-Y386K, and rGIII/A372P-Y386R, were constructed on the basis of the rGIII backbone by replacement of Ala with Ile, Leu, Gly, or Pro at NS5-372, and of Tyr with Lys or Arg at NS5-386.

**Table 3:** Information of amino acids for substitution at NS5-372 and NS5-386.

		Number of hydrogen bonds formed		
Amino acid	Group	NS5-372	NS5-386	Substitution
Val (V)	Nonpolar	2	/	
Gly (G)	Nonpolar	2	/	To replace V or A at NS5-372
Pro (P)	Nonpolar	1	/	To replace V or A at NS5-372
Ala (A)	Nonpolar	3	/	
Ile (I)	Nonpolar	3	/	To replace V or A at NS5-372
Leu (L)	Nonpolar	3	/	To replace V or A at NS5-372
His (H)	Polar Basic	/	1	
Arg (R)	Polar Basic	/	1	To replace H or Y at NS5-386
Lys (K)	Polar Basic	/	1	To replace H or Y at NS5-386
Tyr (Y)	Polar	/	2	To replace H at NS5-386

DEF were infected with these substitution mutant viruses to examine the effects on IFN-α and β induction. The rGI/V372G-H386K, rGI/V372P-H386R, rGIII/A372G-Y386K, and rGIII/A372P-Y386R with one/two hydrogen bonds at NS5-372 and one hydrogen bond at NS5-386 induced levels of IFN-α and β expression similar to those induced by rGI but were significantly lower than those induced by rGIII as well as rGI/V372I-H386Y, rGI/V372L-H386Y, rGIII/A372I, and rGIII/A372L with three hydrogen bonds at NS5-372 and two hydrogen bonds at NS5-386 (Fig 9B). In contrast, the levels of IFN-α and β expression induced by rGI/V372I-H386Y, rGI/V372L-H386Y, rGIII/A372I, and rGIII/A372L were similar to those induced by rGIII but were markedly higher than those induced by rGI as well as rGI/V372G-H386K, rGI/V372P-H386R, rGIII/A372G-Y386K, and rGIII/A372P-Y386R (Fig 9B). These data suggested that the number of hydrogen bonds formed by NS5-372 and NS5-386 contributed to the differences in IFN-α and β induction between the strains, and GI strains with two hydrogen bonds at NS5-372 and one hydrogen bond at NS5-386 induced lower levels of IFN-α and β expression than GIII strains with three hydrogen bonds at NS5-372 and two hydrogen bonds at NS5-386.

Because the number of hydrogen bonds formed by NS5-372 and NS5-386 contributed to the differences in IFN-α and β induction, we examined the effects of the number of hydrogen bonds formed by NS5-372 and NS5-386 on viral replication. DEF were infected with these substitution mutant viruses, and the replication kinetics was measured. The replication titers of rGI/V372G-H386K, rGI/V372P-H386R, rGIII/A372G-Y386K, and rGIII/A372P-Y386R were similar to those of rGI but were significantly higher than those of rGIII as well as rGI/V372I-H386Y, rGI/V372L-H386Y, rGIII/A372I, and rGIII/A372L from 36 to 84 hpi (Fig 9C). In contrast, rGI/V372I-H386Y, rGI/V372L-H386Y, rGIII/A372I, and rGIII/A372L produced viral titers similar to those of rGIII, but were markedly lower than those of rGI as well as rGI/V372G-H386K, rGI/V372P-H386R, rGIII/A372G-Y386K, and rGIII/A372P-Y386R from 36 to 84 hpi (Fig 9C). Additionally, the plaque sizes formed by rGI/V372G-H386K, rGI/V372P-H386R, rGIII/A372G-Y386K, and rGIII/A372P-Y386R were similar to those formed by rGI but clearly larger than those formed by rGIII as well as rGI/V372I-H386Y, rGI/V372L-H386Y, rGIII/A372I, and rGIII/A372L (Fig 9D and 9E). The plaque sizes (S6E Fig) and replication titers (S6F Fig) of all the substitution mutant viruses were tested in BHK cells, and no significant differences were observed. These data suggested that the number of hydrogen bonds formed by NS5-372 and NS5-386 contributed to the differences in replication efficiency between the GI and GIII strains in DEF, and GI strains with two hydrogen bonds at NS5-372 and one hydrogen bond at NS5-386 replicated more efficiently than GIII strains with three hydrogen bonds at NS5-372 and two hydrogen bonds at NS5-386.

### NS5-V372A and NS5-H386Y variations contribute to the differences in IFN-α and β induction and replication efficiency between the GI and GIII strains in ducklings

**Fig 10:**
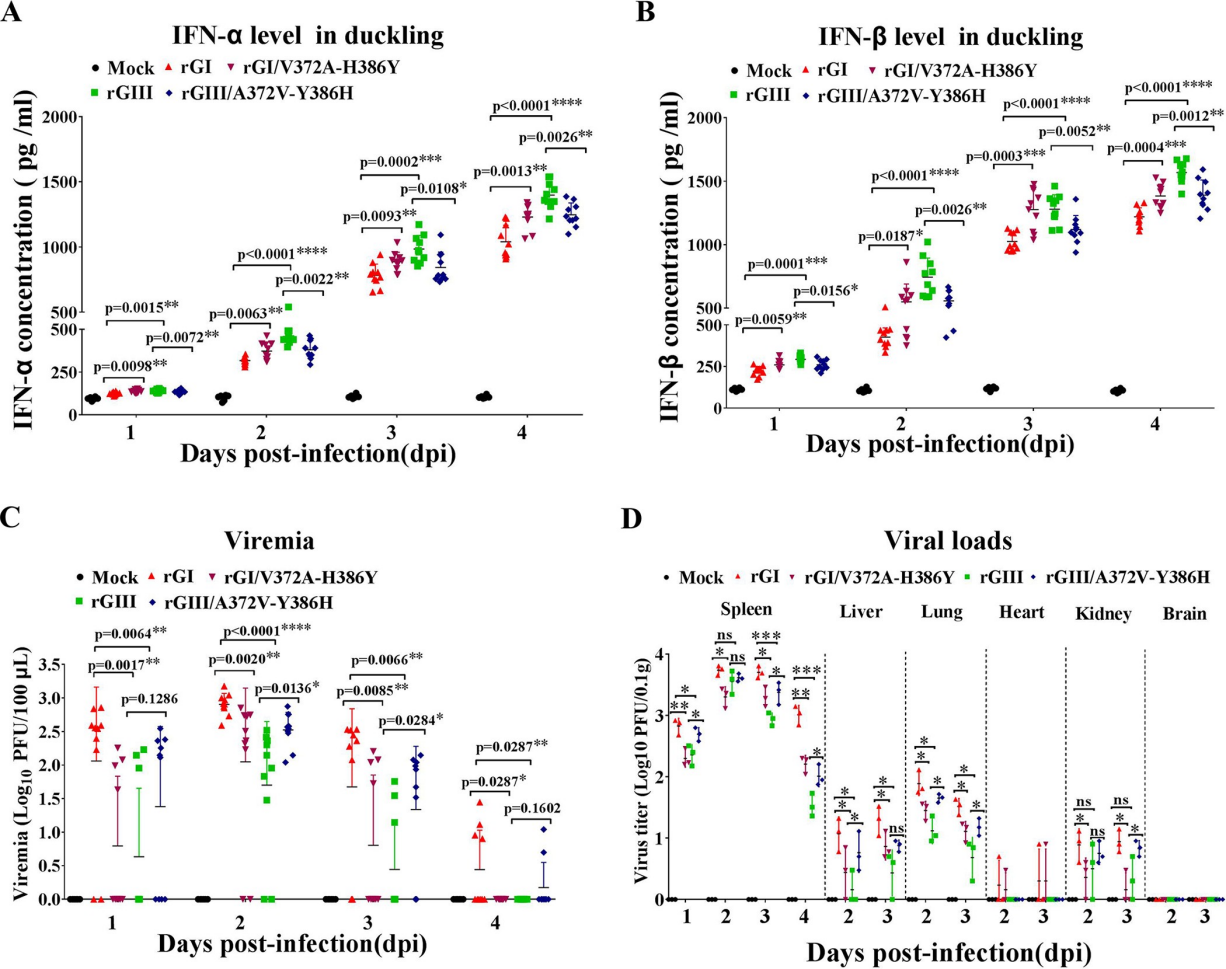
Effects of NS5-V372A and NS5-H386Y variations on IFN-α and β production and viremia in ducklings. (A, B and C) Two-day-old SPF domestic ducklings (*n* = 10) were intramuscularly inoculated with the indicated substitution mutant viruses. Blood samples were collected daily through the jugular vein from 1 to 4 dpi for measurement of the levels of IFN-α (A) and IFN-β (B) production and viremia (C). (D) Detection of viral loads in tissues. Two-day-old SPF domestic ducklings (*n* = 12) were intramuscularly inoculated with the indicated substitution mutant viruses. Three ducklings per group were euthanized daily from 1 to 4 dpi for collection of tissues (spleen, lung, kidney, liver, heart, and brain). Viral loads in each tissue were titrated with TCID_50_ assays on BHK cells. Data are shown as means ± SD. ***, *p* < 0.001; **, *p* < 0.01; *, *p* < 0.05; ns, no significance, by Student’s *t*-test.

Given that NS5-V372A and NS5-H386Y variations contributed to the differences in IFN-α and β induction and replication efficiency between the GI and GIII strains in DEF, we examined the effects of NS5-V372A and NS5-H386Y variations on IFN-α and β production and viral replication in domestic ducklings, which have been used as an avian animal model for experimental infection with JEV[16, 22, 23]. Two-day-old specific pathogen free (SPF) domestic ducklings were subcutaneously inoculated with the substitution mutant virus rGI/V372A-H386Y and rGIII/A372V-Y386H as well as the respective parental rGI and rGIII. Blood samples of the inoculated ducklings were collected daily from 1 to 4 days post-infection (dpi), and the levels of IFN-α and β production and viremia were measured. In response to JEV infection, the levels of IFN-α and β production in all JEV-inoculated ducklings were higher than those in mock-inoculated ducklings (Fig 10A and 10B). Notably, the levels of IFN-α and β production in rGI-inoculated ducklings were significantly lower than those in rGIII-inoculated ducklings, in agreement with the *in vitro* data observed in DEF and DEK cells (Figs 1E and 1F and S2C and S2D), thus suggesting that GI strains induced lower levels of IFN-α and β production than GIII strains.

Comparison of the levels of IFN-α and β production among groups of JEV-inoculated ducklings revealed that the ducklings inoculated with rGI/V372A-H386Y and rGIII/A372V-Y386H showed a trend in IFN-α and β levels opposite from those in ducklings inoculated with the respective parental viruses. rGI/V372A-H386Y with a Val to Ala substitution at NS5-372 plus a His to Tyr substitution at NS5-386 induced significantly higher levels of IFN-α and β production than the parental rGI, whereas the rGIII/A372V-Y386H with an Ala to Val substitution at NS5-372 plus a Tyr to His substitution at NS5-386 elicited clearly lower levels of IFN-α and β production than the parental rGIII (Fig 10A and 10B). These data demonstrated that NS5-V372A and NS5-H386Y variations were responsible for the differences in IFN-α and β induction between the GI and GIII strains in ducklings, and GI strains induced lower levels of IFN-α and β production than GIII strains.

We subsequently measured the levels of viremia in JEV-inoculated ducklings. JEV viremia was detectable as early as 1 dpi, with a viremic rate of 80% (8/10) in rGI-inoculated ducklings, followed by 60% (6/10) in ducklings inoculated with rGI/V372A-H386Y, 40% (4/10) in ducklings inoculated with rGIII/A372V-Y386H, and 30% (3/10) in ducklings inoculated with rGIII. The viremia peaked at 2 dpi and gradually declined over the next few days, and the viremia in rGIII- and rGI/V372A-H386Y-inoculated ducklings disappeared at 4 dpi, whereas a fraction of ducklings inoculated with rGI and rGIII/A372V-Y386H remained viremic (Fig 10C).

The levels of viremia in rGI-inoculated ducklings were significantly higher than those in rGIII-inoculated ducklings during the experimental period (Fig 10C), thus suggesting that rGI replicated more efficiently than rGIII in ducklings, in agreement with previous observations[22, 23]. A significant difference in viremia levels between the ducklings inoculated with the substitution mutant virus and the parental virus was observed. The ducklings inoculated with rGI/V372A-H386Y produced 0.38–1.19 log lower viremia levels than the ducklings inoculated with the parental rGI, and a significant difference was observed at 1 (*p* = 0.0017), 2 (*p* = 0.0020), 3 (*p* = 0.0085), and 4 dpi (*p* = 0.0287). rGIII/A372V-Y386H caused 0.12–0.87 log higher viremia levels in the inoculated ducklings than the parental rGIII, and a significant difference was observed at 2 (*p* = 0.0163) and 3 dpi (*p* = 0.0284) (Fig 10C).

Analysis of viral loads in different tissues indicated that JEV was detectable in the spleens, livers, lungs, hearts, and kidneys, but not in the brains, and the spleens maintained relatively higher viral titers than the other tissues tested (Fig 10D). The viral loads in the spleens of rGI-inoculated ducklings were significantly higher than those in rGIII-inoculated ducklings at 1, 3, and 4 dpi, thus further demonstrating that rGI replicated more efficiently than rGIII. The viral loads in the spleens, kidneys, livers, and lungs differed between ducklings inoculated with the substitution mutant virus versus the parental virus (Fig 10D). The viral titers in the spleens of ducklings inoculated with rGI/V372A-H386Y were significantly lower than those in ducklings inoculated with the parental rGI from 1 to 4 dpi. In contrast, the ducklings inoculated with rGIII/A372V-Y386H showed markedly higher viral loads in the spleens than those in ducklings inoculated with the parental rGIII at 1, 3, and 4 dpi. Similar results were also observed in the livers, lungs, and kidneys (Fig 10D). In addition, JEV was detectable in the hearts of a few ducklings inoculated with rGI and rGIII/A372V-Y386H, but not in the ducklings inoculated with rGIII and rGI/V372A-H386Y (Fig 10D). Together, these data demonstrated that NS5-V372A and NS5-H386Y variations contributed to the differences in replication efficiency between the GI and GIII strains in ducklings, and the GI strains replicated more efficiently than GIII strains.

## Discussion

GI strains replicate more efficiently than GIII strains in birds, showing strain-specific differences in host adaptability that are considered to contribute to the JEV genotype shift[22, 23]. The differences in replication efficiency in birds has been speculated to be a result of differing antagonism of the IFN-I mediated antiviral response in the GI and GIII strains[22]. In this study, we observed the differences in IFN-α and β induction between the strains in both DEF and domestic ducklings, and identified the NS5-V372A and NS5-H386Y variations responsible for the differences in IFN-α and β induction, which thereby resulted in the differences in replication efficiency between the GI and GIII strains in birds.

We observed that GI strains induced lower levels of IFN-α and β production than GIII strains in duck-derived DEF and DEK cells, but not in pig-derived ST and PIEC cells and mouse-derived MEF and bEnd.3 cells, thus indicating host specificity. The ability of GI strains to induce low levels of IFN-α and β production was further confirmed in domestic ducklings, an avian animal model for experimental infection with JEV[16, 22, 23]. In addition, the ability of GI strains to induce low levels of IFN-α and β production was associated with the higher replication efficiency of GI strains than GIII strains. Overall, these observations demonstrated that GI strains, compared with GIII strains, induced low levels of IFN-α and β production for antagonism of the IFN-I mediated antiviral response, thus resulting in enhanced replication efficiency in birds, and showing an enhanced host adaptability of GI strains over GIII strains in birds.

Using a series of chimeric recombinant viruses with exchange of structural and non-structural proteins between the GI and GIII strains, we identified NS5 as the viral determinant of the differences in IFN-α and β induction and replication efficiency between the strains in DEF and ducklings. NS5 is known to play a role in inhibition of IFN-I production during JEV infection[36] and has been speculated to be involved in the JEV genotype shift[9]. Comparing the effects of NS5 on inhibition of IFN-α and β production between the GI and GIII strains, we observed that NS5 of GI strains inhibited the IFN-α and β expression more efficiently than NS5 of GIII strains in DEF stimulated with poly(I:C). On the basis of these findings, we concluded that the greater inhibitory effect of NS5 of GI strains on IFN-α and β expression resulted in the lower levels of IFN-α and β induction and the higher levels of replication efficiency of GI strains than GIII strains, which might contribute to the enhanced host adaptability of GI strains over GIII strains in birds and thereby play a role in the JEV genotype shift. Indeed, NS5 has been demonstrated to antagonize the IFN-I mediated immune response in a host-specific manner and to play a role in determining the host range of flavivirus. For example, NS5 of DENV, ZIKV, and yellow fever virus binds primate signal transducer and activator of transcription 2 (Stat2) and antagonizes IFN signaling, but is unable to bind mouse Stat2 and antagonize murine IFN signaling[25, 41, 42].

Although the exogenous expression of NS5 inhibited both IFN-α and β expression, a linear dose dependency was observed between the reduction of IFN-β expression and the increase of NS5 expression, but not between the reduction of IFN-α expression and the increase of NS5 expression. This apparent difference may be attributable to the differences in regulation of gene expression between IFN-α and IFN-β. It is known that IFN-α and β production is primarily controlled at the gene transcriptional level upon viral infection. A family of transcription factors including interferon regulatory factors (IRFs) plays central roles in activation of IFN-α and β gene transcription[43]. For example, IFN-α expression is completely abolished, and the expression of IFN-β is markedly inhibited in IRF7 knockout mice in response to viral infections. In contrast, in IRF3 knockout mice, the expression of IFN-α was not affected, whereas IFN-β was moderately inhibited[44]. NS5 may interact with different molecules involved in regulation of IFN-α and β expression to impair IFN-α and β production. However, the exact mechanisms underlying the differences in inhibition of IFN-α and β expression by NS5 is needed to be explored.

NS5 was found to contain a total of 11 amino acid variations between the GI and GIII strains. With a series of chimeric and substitution mutant viruses, we identified that NS5-V372A and NS5-H386Y variations co-contribute to the differences in IFN-α and β induction and replication efficiency between the GI and GIII strains in DEF and ducklings. Either a Val to Ala substitution at NS5-372 or a His to Tyr substitution at NS5-386 impaired the ability of GI strains to induce low levels of IFN-α and β and produce high replication titers. In contrast, an Ala to Val substitution at NS5-372 plus a Tyr to His substitution at NS5-386 enhanced the ability of GIII strains to induce low levels of IFN-α and β and produce high replication titers. These findings demonstrated that NS5-V372A and NS5-H386Y variations co-contributed to the differences in host adaptability between the GI and GIII strains in birds, thereby playing a role in the JEV genotype shift. In addition, these findings provided experimental evidence suggesting that NS5-H386Y is a site under positive selection that may be associated with host adaption during the JEV genotype shift[9].

NS5 in flavivirus is a conformationally variable protein[45, 46] that exhibits a high degree of flexibility in solution and is able to adopt distinct conformations, thus substantially affecting its diverse functions[40, 47]. The NS5-V372A and NS5-H386Y variations were located in the α-helix of the NLS region of NS5, which is involved in interaction with importins, antagonism of IFN-I production, and viral replication[36, 38]. Structurally, NS5-372 and NS5-386 in GI strains formed fewer hydrogen bonds with their neighboring residues than in GIII strains, thus potentially resulting in the differences in the flexibility of the NLS region between the GI and GIII strains and thereby leading to changes in interactions with host cell proteins and subsequent IFN-I production and viral replication. In fact, the substitution analysis indicated that the number of hydrogen bonds formed by NS5-372 and NS5-386 contributed to the differences in IFN-α and β induction and replication efficiency between the GI and GIII strains, and GI strains with two hydrogen bonds at NS5-372 and one hydrogen bond at NS5-386 induced lower levels of IFN-α and β expression and replicated more efficiently than GIII strains with three hydrogen bonds at NS5-372 and two hydrogen bonds at NS5-386. Apart from the structural flexibility of the NLS region of NS5, no significant differences in IFN-α and β induction were observed between rGI/V372G-H386K and rGI/V372P-H386R, which formed three and two hydrogen bonds with neighboring residues, respectively. Similar results were also observed between rGIII/A372G-Y386K and rGIII/A372P-Y386R. These data implied that an alternative molecular basis underlying the inhibition of IFN-α and β induction by NS5 may present.

NS5 in flavivirus is the largest viral protein consisting of MTase, N-ext, and RdRp domains [37], and it plays a critical role as an IFN antagonist in the antagonism of the IFN-I mediated antiviral response via different mechanisms. For example, NS5 of DENV and ZIKV binds and depletes Stat2 via ubiquitin-dependent proteasomal degradation, thus suppressing the activation of IFN-I-stimulated Jak-Stat signaling in primates[48, 49]. NS5 of WNV and tick-borne encephalitis virus suppresses the maturation and cell surface expression of the IFN-I receptor via interaction with the host protein prolidase, thus preventing IFN-I downstream signaling and the expression of IFN stimulated genes[48]. In the case of JEV, NS5 readily blocks IFN-stimulated Jak-Stat signaling events, such as Stat1 nuclear translocation and tyrosine phosphorylation of Tyk2 and Stat1 in mammalian cells[50]. In addition, NS5 of JEV binds importin-α3 and importin-α4 via its NLS region, thus competitively blocking the interaction of importin-α3 and importin-α4 with their cargo molecules IRF3 and p65, and thereby resulting in inhibition of the nuclear translocation of IRF3 and NF-κB and subsequent IFN-I production. Mutagenesis of key residues in the NLS region impairs the interaction of NS5 with importins and restores expression of IFN stimulated genes[36]. According to these previous observations, we speculated that NS5-V372A and NS5-H386Y variations present in the NLS region might alter the flexibility of the NLS region, thus altering the ability of NS5 to interact with avian cell proteins involved in the IFN-I signaling pathway, such as importins, and consequently affecting IFN-I production in birds. However, all these previous findings related to antagonism of IFN-I mediated antiviral response were observed in mammalian cells, and we were unable to test this possibility in avian cells in this study, because of the limited information available on the avian IFN-I signaling pathway, which is largely unknown and cannot be simply extrapolated from human and mouse data[51].

The differences in IFN-α and β induction between the GI and GIII strains was observed in duck-derived DEF, but not in pig-derived ST cells and mouse-derived bEnd.3 cells, thus indicating host specificity. Flavivirus has evolved different mechanisms to adapt to the antiviral immune response of distinct host species. For example, WNV, which belongs to the same evolutionary clade and shares same mosquito vectors and vertebrate zoonotic amplifying hosts (birds) with JEV, is able to adapt its immune restriction mechanisms to insects, birds, and mammals[52]. Birds have a smaller repertoire of immune genes than mammals and lack some key genes responsible for antiviral immune response[51]. For example, IRF3, an essential transcriptional regulator of IFN genes, is absent in birds[53], and retinoic acid-inducible gene-I, which is the intracellular detector of single-stranded viral RNA, is absent in chickens[54, 55]. Therefore, birds, compared with mammals, have impaired detection of viruses and intracellular pathogens [51]. The differences in IFN-α and β induction between the GI and GIII strains observed only in duck-derived DEF and ducklings, but not in pig-derived ST cells and mouse-derived bEnd.3 cells, might be attributable to the differences in adaptability of GI and GIII strains to the IFN-I mediated antiviral response of birds and mammals, and to the differences in the antiviral immune response between birds and mammals. The NS5-V372A and NS5-H386Y variations enabled GI strains to adapt to the IFN-I mediated antiviral response of birds, but not mammals, thereby leading to replication and host adaption advantages of GI strains over GIII strains in birds. This finding might explain the host-specific differences in IFN-I induction between the GI and GIII strains in different hosts.

The host adaptability of flavivirus is associated with genetic changes in multiple genes[26, 56–59]. An evolutionary mutation in NS1 enhances the ability of ZIKV to infect mosquitoes and inhibit IFN-β induction in mammals[26]. Amino acid changes in the E protein of duck tembusu virus, a newly emerging pathogenic flavivirus, are responsible for tissue tropism and transmissibility in ducks[60]. Mutation and selection pressure analysis of GI and GIII strains suggests that other viral proteins, such as E and NS4B, harboring amino acid variations, in addition to NS5, may contribute to the replication and host adaption advantages of GI strains over GIII strains in hosts[9, 61]. In the present study, the identification of NS5 as being responsible for the differences in replication efficiency between the GI and GIII strains was based on the differences in IFN-α and β induction. Other mutated proteins or nucleotides might possibly also contribute to the differences in replication efficiency and host adaptability between the GI and GIII strains. Indeed, NS2B/NS3 mutations have recently been demonstrated to contribute to the enhanced infectivity of GI strains over GIII strains in pigs, ducklings, and young chickens[22].

In summary, we examined the ability of the GI and GIII strains to induce IFN-α and β production and observed a significant difference in induction of IFN-α and β production between the GI and GIII strains in duck-derived DEF and ducklings. GI strains induced lower levels of IFN-α and β production, which were corelated with higher replication efficiency in GI strains than GIII strains. The NS5-V372A and NS5-H386Y variations were identified to together contribute to the differences in IFN-α and β induction and the replication efficiency between the strains. The NS5-V372A and NS5-H386Y variations enabled GI strains to inhibit IFN-α and β production more efficiently than GIII strains for antagonism of the IFN-I mediated antiviral response, thereby leading to the replication and host adaption advantages of GI strains over GIII strains in birds. A limitation of this study is that we were unable to explore the mechanisms of how NS5-V372A and NS5-H386Y variations enable GI strains to inhibit IFN-α and β production more efficiently than GIII strains in birds, because of the limited knowledge of the IFN-I mediated antiviral response in birds. However, we identified the NS5-V372A and NS5-H386Y variations as the viral determinant responsible for the differences in IFN-α and β induction and replication efficiency between the GI and III strains in birds, thus providing new insight into the molecular basis of the JEV genotype shift.

## Materials and methods

### Ethics statement

All animal experiments were approved by the Institutional Animal Care and Use Committee of Shanghai Veterinary Research Institute (IACUC No: SHVRI-SZ-2019050402) and were performed in compliance with the Guidelines on the Humane Treatment of Laboratory Animals (Ministry of Science and Technology of the People’s Republic of China, Policy No. 2006 398).

### Cells and antibodies

DEF and DEK prepared from 11- to 13-day-old SPF duck embryos, BHK, ST, PIEC, MEF, and bEnd.3 cells were cultured in Dulbecco’s modified Eagle’s medium containing 10% fetal bovine serum (FBS; Thermo Fisher Scientific, Waltham, MA, USA), 100 μg/ml streptomycin, and 100 IU/ml penicillin at 37°C. The C6/36 cells were cultured in RPMI medium 1640 (Thermo Fisher Scientific) containing 10% FBS, 100 μg/ml streptomycin and 100 IU/ml penicillin at 28°C. The commercial antibodies used in this study included an anti-NS5 polyclonal antibody (GeneTex, St. Anthony, TX, USA), an anti-actin monoclonal antibody (Proteintech, Chicago, IL, USA), an anti-Flag monoclonal antibody (M2; Sigma, St. Louis, MO, USA), a fluorescein isothiocyanate-conjugated goat anti-mouse IgG antibody (Thermo Fisher Scientific), and horseradish peroxidase-conjugated goat anti-mouse IgG antibody (Abcam, Shanghai, China).

### Preparation of virus stocks

The GI strains of SH7 (GenBank No. MH753129) and SD12 (GenBank No. MH753127), and GIII strains of SH15 (GenBank No. MH753130) and SH19 (GenBank No. MH753131) used in this study were previously isolated from aborted pigs or mosquitoes during 2015–2016[29]. All JEV strains were plaque-purified three times in BHK cells and subsequently amplified in BHK cells at 0.1 MOI. The supernatants collected from the infected BHK cells were subjected to sucrose gradient ultracentrifugation, as described previously[62]. The white matter of fraction containing JEV was collected and diluted with phosphate buffered saline (PBS) in an ultracentrifuge tube. After centrifugation at 200,000 x g at 4°C for 1.5 h, the pellet was resuspended in PBS and dissolved overnight at 4°C. The virus stocks were stored at −80°C until use and the viral titer was measured in BHK cells. To inactivate JEV, virus was irradiated using a CL-1000 UV crosslinker (UVP, California, USA) at 500 mJ/cm^2^ for 15 min. The inactivation was confirmed by inoculation of the irradiated virus into BHK cells.

### Generation of chimeric recombinant viruses

Viral RNA extraction of SH7 and SH15 strains for synthesis of cDNA was performed as described previously[63]. Four pairs of primers targeting the highly conserved sequences of GI and GIII strains (S3 Table) were used to amplify four overlapping fragments covering the full-length cDNA of the SH7 and SH15 strains, respectively. The fragments amplified by PCR were inserted into the pOK12 vector to generate four recombinant plasmids containing differences in fragments in each strain. The generated recombinant plasmids of the SH7 strain were pSH7T7-1-2361 (from nucleotides 1 to 2361 with the addition of a T7 promoter at the 5´ terminus), pSH7-2332-4497 (from nucleotides 2332 to 4497), pSH7-4468-7664 (from nucleotides 4468 to 7664), and pSH7-7636-10965 (from nucleotides 7636 to 10965). The generated recombinant plasmids of the SH15 strain included pSH15T7-1-2360 (from nucleotides 1 to 2332 with the addition of a T7 promoter at the 5´ terminus), pSH15-2331-4496 (from nucleotides 2331 to 4496), pSH15-4467-7663 (from nucleotides 4467 to 7663), and pSH15-7635-10977 (from nucleotides 7635 to 10977). The full-length infectious cDNA clone of each strain was generated *in vitro* by splicing of the four fragments through homologous recombination with a Gibson Assembly Cloning Kit (New England Biolabs, Ipswich, MA, USA). The plasmids for substitution of viral proteins were constructed by homologous recombination. The plasmids for point mutations at NS5-372 and NS5-386 were generated with PCR-based site-directed mutagenesis of plasmid pSH7-7636-10965 or pSH15-7635-10977. The primers used are listed in S3 Table. Full-length viral RNA was transcribed *in vitro* from the full-length viral cDNA template with a mMessage mMachine T7 kit (Invitrogen, Carlsbad, CA, USA). The generated viral RNA transcripts were treated with DNase I to remove the cDNA template and subsequently transfected into BHK cells on a six-well plate with DMRIE-C reagent (Invitrogen) according to the manufacturer’s instructions. Supernatants of the transfectants showing the cytopathic effects of JEV were collected and amplified in BHK cells. All recombinant viruses rescued from BHK cells were plaque-purified three times in BHK cells and subsequently confirmed by Sanger sequencing by Invitrogen Corporation (Shanghai, China) (S8 Fig). The clones of recombinant viruses harboring any unwanted mutation were discarded.

### Growth kinetics and viral plaque assays

Cells were pre-cultured in 96-well plates to 80% confluence and infected with JEV at 0.01 or 0.1 MOI. After 1 h incubation, the inoculants were discarded, and the cells were washed three times with PBS, and fresh Dulbecco’s modified Eagle’s medium with 2% FBS was then added. Supernatants of the JEV-infected cells were sampled at different time points and titrated with TCID_50_ assays on BHK cells. For viral plaque assays, DEF or BHK cells were infected with JEV at 100 plaque forming units (PFU) and incubated for 2 h at 37°C. The cells were washed three times with PBS and then cultured in modified Eagle’s medium (MEM) containing 2% FBS and 1% agarose for 4 days. The cells were fixed with 4% paraformaldehyde for 2 h at room temperature, and the viral plaques were stained with 0.5% crystal violet.

### Detection of viral RNA copies by TaqMan probe-based qRT-PCR

Total RNA was extracted from JEV-infected cells using TRIzol reagent (Thermo Fisher Scientific) following the manufacturer’s instructions. Reverse transcription was performed using Superscript II reverse transcriptase (Invitrogen) according to the manufacturer's instructions. The copies of viral PrM/M gene were determined using TaqMan probe-based qRT-PCR, as described previously[64].

### Detection of IFN-α and β expression by qRT-PCR

Cells were infected with JEV at 0.1 or 1 MOI and collected at the indicated time points. Total RNA was extracted from cell samples with TRIzol reagent (Thermo Fisher Scientific) according to the manufacturer's instructions. Reverse transcription was performed with a PrimeScript RT reagent kit with gDNA Eraser (TaKaRa, Kyoto, Japan). The mRNA levels of IFN-α and β were determined with qRT-PCR with SYBR Premix Ex Taq (TaKaRa) and normalized to the mRNA levels of the internal control glyceraldehyde-3-phosphate dehydrogenase. Fold change in gene expression was calculated with the 2^-ΔΔCT^ method. Primers used are shown in S3 Table.

### Measurement of IFN-α and β concentrations

Supernatants of the JEV-infected cells were collected at different time points and subjected to measurement of IFN-α and β protein concentrations with ELISA kits according to the manufacturer’s instructions. The ELISA kits for detection of pig IFN-α (No. CSB-E07328p) and IFN-β (No. CSB-E09890p), mouse IFN-α (No. CSB-E08638m) and IFN-β (No.CSB-E04945m) were purchased from Cusabio (CUSABIO, Wu Han, China). The ELISA kits for detection of duck IFN-α (No. BPE60121) and IFN-β (No. BPE60092) were purchased from Lengton Bioscienc Co., LTD (Lengton, Shanghai, China).

### Exogenous expression of Flag-NS5 and poly(I:C) treatment

Plasmids engineered to express Flag-NS5 were constructed by inserting NS5 cDNA into p3xFlag-CMV-7.1 (Sigma). Flag-NS5 mutant was generated by PCR-based site-directed mutagenesis. Cells were transfected with plasmids expressing Flag-NS5 using Lipofectamine^TM^ 3000 Transfection Reagent (Thermo Fisher Scientific) according to the manufacturer’s instructions, and incubated at 37°C for 24 h. The transfectants were treated with poly(I:C) (1μg/mL) (Sigma) and incubated at 37°C for an additional 12 h. Flag-NS5 expression was detected by western blotting with anti-Flag antibody. IFN-α and β production were measured at the mRNA levels by qRT-PCR and protein levels by ELISA.

### Western blot and immunofluorescence assays

DEF were infected with JEV or transfected with plasmids for expression of NS5 and incubated for 24 h. JEV replication and NS5 expression were detected by western blotting with anti-NS5 antibodies and anti-Flag antibodies, respectively. DEF pre-cultured on cover slips were infected with JEV and incubated for 24 h. The cells were fixed in 4% paraformaldehyde for immunofluorescence assays with anti-NS5 antibodies. Western blot analysis and immunofluorescence assays were performed as previously described[65].

### RNA interference

The siRNA-269 (5′-UCCAACACCUCUUCGACACTT-3′) and siRNA-425 (5′-GCAACCUUCACCUCAGCAUTT-3′) targeting duck IFN-α mRNA (GenBank no. AB128861.1), and siRNA-272 (5′-UCCAACACCUCUUCAACAUTT3′) and siRNA-464 (5′-GCAUCCAACACUUCCUCCATT-3′) targeting duck IFN-β mRNA (GenBank no. KM035791.2) were designed using GenScript (https://www.genscript.com/ssl-bin/app/rnai) and RNA wizard (http://www.sirnawizard.com/design_advanced.php), and blasted against the reference sequence database of duck to reduce a risk of silencing unintended genes. The siRNA-269, siRNA-425, siRNA-272, siRNA-464, and scrambled RNA control were chemically synthesized (GenePharma, Shanghai, China) and used for silencing duck IFN-α and β expression. DEF was transfected with a combination of siRNA-269 and siRNA-425, or siRNA-272 and siRNA-464 using Lipofectamine RNAi MAX (Thermo Fisher Scientific) and incubated for 12 h. The transfectants were infected with JEV at 0.1 MOI and harvested at 24 and 36 hpi for measurement of IFN-α and β expression and viral replication.

### Animal experiments

Two-day-old SPF domestic ducklings were randomly divided into four groups (*n* = 10), which were intramuscularly mock-inoculated with PBS or inoculated with 10,000 PFU of recombinant JEV. Blood samples were collected daily via the jugular vein from 1 to 4 dpi for detection of IFN-α and β production and viremia. For detection of viral loads in tissues, 2-day-old SPF domestic ducklings were inoculated intramuscularly with 10,000 PFU of recombinant JEV. Three of the inoculated ducklings per group were euthanized daily from 1 to 4 dpi. Tissues including spleens, lungs, kidneys, livers, hearts, and brains were collected from the euthanized ducklings for detection of viral load.

### Detection of viremia and viral load in tissues

Blood samples collected from JEV-inoculated ducklings were serially 10-fold diluted in MEM and inoculated in BHK cells for detection of viremia with TCID_50_ assays, as described previously[23]. Tissue samples collected from JEV-inoculated ducklings were homogenized with PBS containing 0.1% penicillin and 0.1% streptomycin and centrifuged, and the supernatants were collected. The collected supernatants were serially 10-fold diluted in MEM and inoculated in BHK cells for detection of viral loads with TCID_50_ assays.

### Phylogenetic analysis and sequence alignment

A total 50 representative GI and GIII strains (S1 Table) were downloaded from NCBI (http://www.ncbi.nlm.nih.gov) and phylogenetically analyzed with MEGA version 6.06 software (https://www.megasoftware.net). The sequence alignment was performed with DNASTAR Software (DNAStar, Madison, WI, USA).

### Homology modeling

The image of NS5 was created with SWISS-MODEL (https://swissmodel.expasy.org/). The NS5 structure from Protein Data Bank accession number 4k6m was visualized and analyzed with PyMOL software (http://www.pymol.org) and SPDBV (DeepView) software (https://spdbv.vital-it.ch). The hydrogen bonds between amino acid residues were analyzed in SPDBV (DeepView) software.

### Statistical analysis

Statistical analysis was performed with GraphPad Prism 7 (GraphPad, La Jolla, CA, USA). Data are expressed as means with standard deviations (SD). Significance was assessed with Student’s *t*-test. A *p* value < 0.05 was considered significant.

## Supporting information

S1 Fig Detection of JEV infection by immunofluorescence assay. DEF, DEK, ST, PIEC, bEnd.3, MEF, and C6/36 cells were infected with SH7, SD12, SH15, and SH19 strains at 0.1 MOI and incubated for 24 h. Expression of viral NS5 was detected with immunofluorescence assay with anti-NS5 antibodies (green). Nuclei were stained with DAPI (blue).
(TIF)

S2 Fig Differences in IFN-α and β induction and replication efficiency between the GI and GIII strains. PIEC, MEF, DEK, and C6/36 cells were infected with GI (SH7 and SD12) and GIII (SH15 and SH19) strains at 0.1 MOI and harvested at 24, 36, and 48 hpi for measurement of IFN-α and β production and viral replication. (A, B and C) The mRNA levels of IFN-α and β in the cell pellets were examined by qRT-PCR. (D) The concentrations of IFN-α and β proteins in the supernatants were determined by ELISA. (E, G, I and K) The replication titers of GI and GIII strains in the supernatants were titrated with TCID_50_ assays in BHK cells and the significant differences between the average titers of GI and GIII strains were tested at each time point. (F, H, J and L) The levels of viral NS5 were examined with western blotting with anti-NS5 antibodies. All data are presented as mean ± SD from three independent experiments. ns, no significant difference by Student’s *t*-test.
(TIF)

S3 Fig Amino acid variations conserved between the GI and GIII strains. Phylogenetic analysis and multiple sequence alignments were determined with MEGA version 6.06 and DNASTAR software, respectively. The number highlighted in black indicates the amino acid residue position. * indicates the strains used in this study.
(TIF)

S4 Fig Detection of IFN-α and β expression and replication efficiency of chimeric recombinant viruses in DEF. (A and B) DEF were infected with the indicated chimeric recombinant viruses at 1 MOI and harvested at 24 hpi for measurement of IFN-α and β expression at the mRNA level by qRT-PCR. All data are presented as mean ± SD from three independent experiments. *, *p* < 0.05; **, *p* < 0.01; ***, *p* < 0.001; ns, no significant difference, by Student’s *t*-test. (A) Chimeric recombinant viruses with exchange of structural proteins. (B) Chimeric recombinant viruses with exchange of non-structural proteins. (C) DEF were infected with the indicated chimeric recombinant viruses at 0.01 MOI and harvested at the indicated time points for measurement of viral replication with TCID_50_ assays in BHK cells. All data are presented as mean ± SD from three independent experiments and were tested by Student’s *t*-test. The significant difference between rGI/SH15CPrME and rGIII/SH7CPrME at different time points is marked (*, *p* < 0.05; **, *p* <0.01). The significant difference between rGI/SH15NS1 and rGIII/SH7NS1 at different time points is labeled (#, *p* < 0.05; ##, *p* < 0.01). The significant difference between rGI/SH15NS2A and rGIII/SH7NS2A at different time points is labeled (&, *p* < 0.05; &&, *p* < 0.01). The significant difference between rGI/SH15NS2B/NS3 and rGIII/SH7NS2B/NS3 at different time points is labeled (φ, *p* < 0.05). The significant difference between rGI/SH15NS4A and rGIII/SH7NS4A at different time points is labeled (δ, *p* < 0.05; δδ, *p* < 0.01). (D and E) Monolayers of DEF were infected with the indicated chimeric viruses and the respective parental viruses at 100 PFU for analysis of plaque morphology. The plaques were stained with crystal violet at 4 dpi (E) and the plaque diameters were measured and plotted (D). The significant differences between groups were tested by Student’s *t*-test (***, *p* < 0.001).
(TIF)

S5 Fig Determination of the RdRp region responsible for the differences in IFN-α and β induction and replication efficiency between the GI and GIII strains. (A, B and C) DEF were infected with the indicated chimeric recombinant viruses at 0.5, 1, and 5 MOI and harvested at 24 hpi for measurement of IFN-α and β expression at the mRNA level by qRT-PCR. All data are presented as mean ± SD from three independent experiments. *, *p* < 0.05; **, *p* < 0.01; ***, *p* <0.001; ns, no significant difference, by Student’s *t*-test. (A) Chimeric recombinant viruses with exchange of MTase, N-ext, and RdRp. (B) Chimeric recombinant viruses with exchange of aRdRp and bRdRp. (C) Chimeric recombinant viruses with exchange of a-aRdRp and b-aRdRp. (D) DEF were infected with the indicated chimeric recombinant viruses at 0.01 MOI and harvested at the indicated time points for measurement of viral replication with TCID_50_ assays in BHK cells. All data are presented as mean ± SD from three independent experiments and were tested by Student’s *t*-test. The significant difference between rGI and rGI/SH15RdRp at different time points is marked (*, *p* < 0.05; **, *p* <0.01). The significant difference between rGI and rGI/SH15aRdRp at different time points is labeled (#, *p* < 0.05).The significant difference between rGIII and rGIII/SH7RdRp at different time points is labeled (&, *p* < 0.05).The significant difference between rGIII and rGIII/SH7aRdRp at different time points is labeled (φφ, *p* < 0.01; φ, *p* < 0.05). (E) Monolayers of DEF were infected with the indicated chimeric viruses and the respective parental viruses at 100 PFU for analysis of plaque morphology. The plaques were stained with crystal violet at 4 dpi.
(TIF)

S6 Fig Replication efficiency of chimeric recombinant viruses in BHK cells. (A, C, and F) BHK cells were infected with the indicated chimeric viruses at 0.01 MOI for analysis of replication efficiency. The supernatants were sampled at the indicated time points and titrated with TCID_50_ assays on BHK cells. (B, D, and E) Monolayers of BHK cells were infected with the indicated chimeric viruses and the respective parental viruses at 100 PFU for analysis of plaque morphology. The plaques were stained with crystal violet at 4 dpi.
(TIF)

S7 Fig Effect of NS5-V372A and NS5-H286Y variations on IFN-α and β induction and replication efficiency of recombinant viruses in ST, bEnd.3 and C6/36 cells. (A and B) ST and bEnd.3 cells were infected with the indicated recombinant viruses at 0.1, 1, and 5 MOI and harvested at 24 hpi for measurement of IFN-α and β production with qR-PCR. (C to H) ST, bEnd.3, and C6/36 cells were infected with the indicated recombinant viruses at a MOI of 0.01 and harvested at the indicated time points for analysis of replication efficiency. The replication titers in the supernatants were titrated with TCID_50_ assays in BHK cells (C, E and G). The levels of NS5 protein in the cells were examined with western blotting with anti-NS5 antibodies (D, F and H). All data are presented as mean ± SD from three independent experiments. ns, no significant difference by Student’s *t*-test.
(TIF)

S8 Fig Sequence data of substitution mutant viruses. (DOCX)

S1 Table Information on the JEV strains used for multiple sequence alignment. (DOCX)

S2 Table Number of hydrogen bonds formed by residues NS5-372 and NS5-386 with neighboring residues. (DOCX)

S3 Table Primers used in the study. (DOCX)
