# Supplementary figures and images for: NS5-V372A and NS5-H386Y variations are responsible for differences in interferon α/β induction and co-contribute to the replication advantage of Japanese encephalitis virus genotype I over genotype III in ducklings
Source: PLoS Pathog. 2020 Sep 3;16(9):e1008773. doi: 10.1371/journal.ppat.1008773 (PMC7494076; doi:10.1371/journal.ppat.1008773)

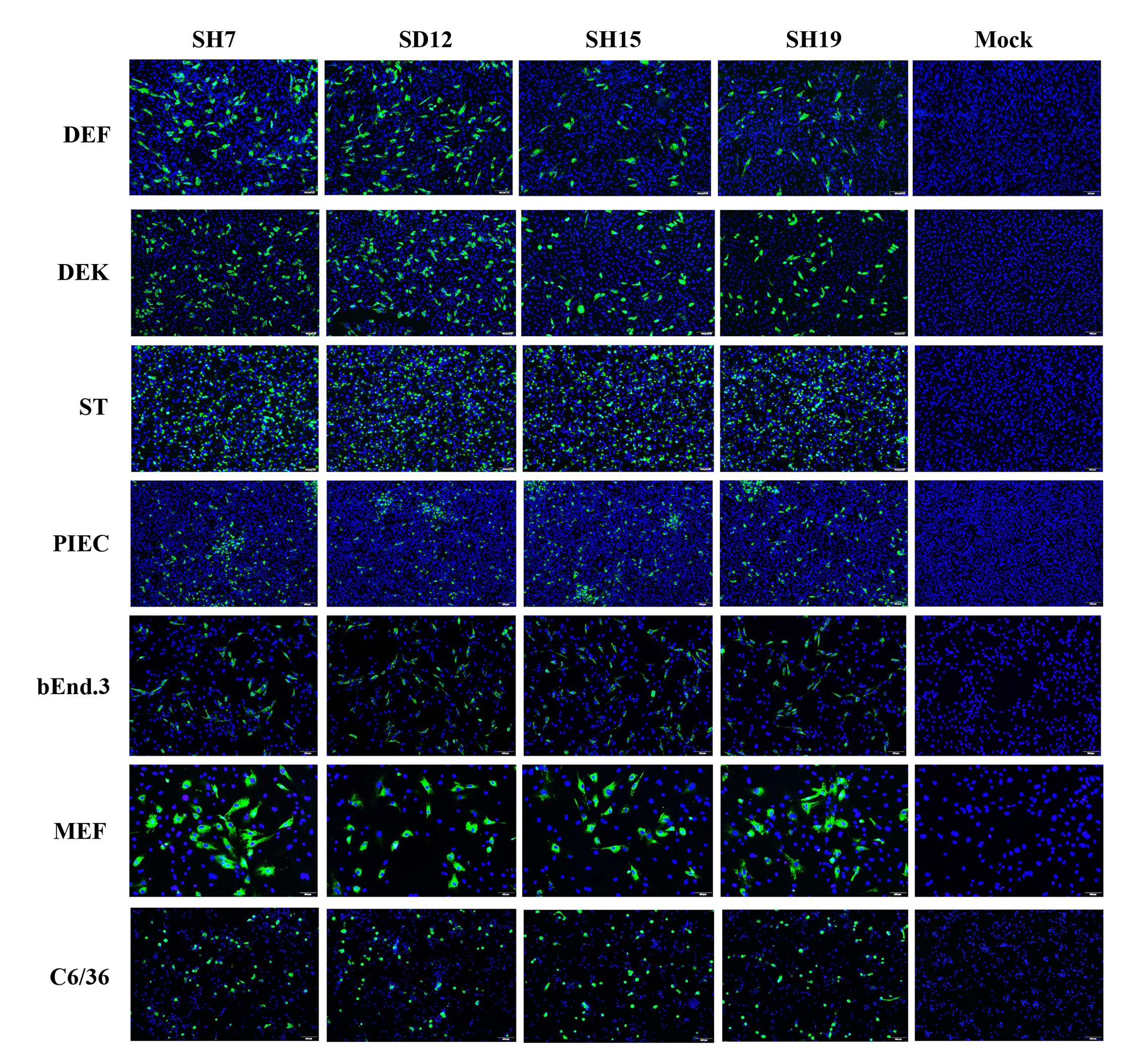

Supplement: S1 Fig — DEF, DEK, ST, PIEC, bEnd.3, MEF, and C6/36 cells were infected with SH7, SD12, SH15, and SH19 strains at 0.1 MOI and incubated for 24 h. Expression of viral NS5 was detected with immunofluorescence assay with anti-NS5 antibodies (green). Nuclei were stained with DAPI (blue). (TIF) [file ppat.1008773.s001.tif]

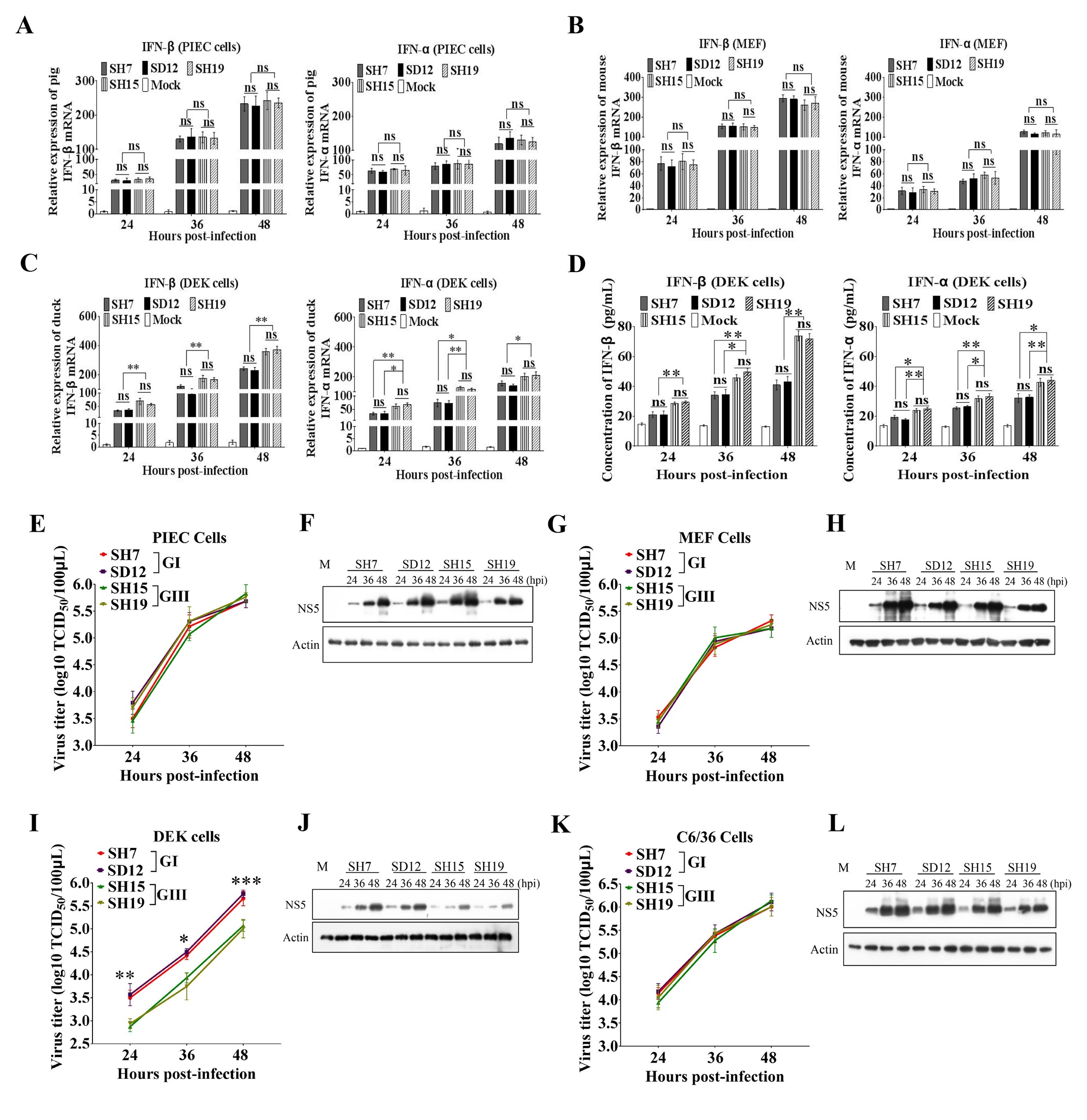

Supplement: S2 Fig — PIEC, MEF, DEK, and C6/36 cells were infected with GI (SH7 and SD12) and GIII (SH15 and SH19) strains at 0.1 MOI and harvested at 24, 36, and 48 hpi for measurement of IFN-α and β production and viral replication. (A, B and C) The mRNA levels of IFN-α and β in the cell pellets were examined by qRT-PCR. (D) The concentrations of IFN-α and β proteins in the supernatants were determined by ELISA. (E, G, I and K) The replication titers of GI and GIII strains in the supernatants were titrated with TCID50 assays in BHK cells and the significant differences between the average titers of GI and GIII strains were tested at each time point. (F, H, J and L) The levels of viral NS5 were examined with western blotting with anti-NS5 antibodies. All data are presented as mean ± SD from three independent experiments. ns, no significant difference by Student’s t-test. (TIF) [file ppat.1008773.s002.tif]

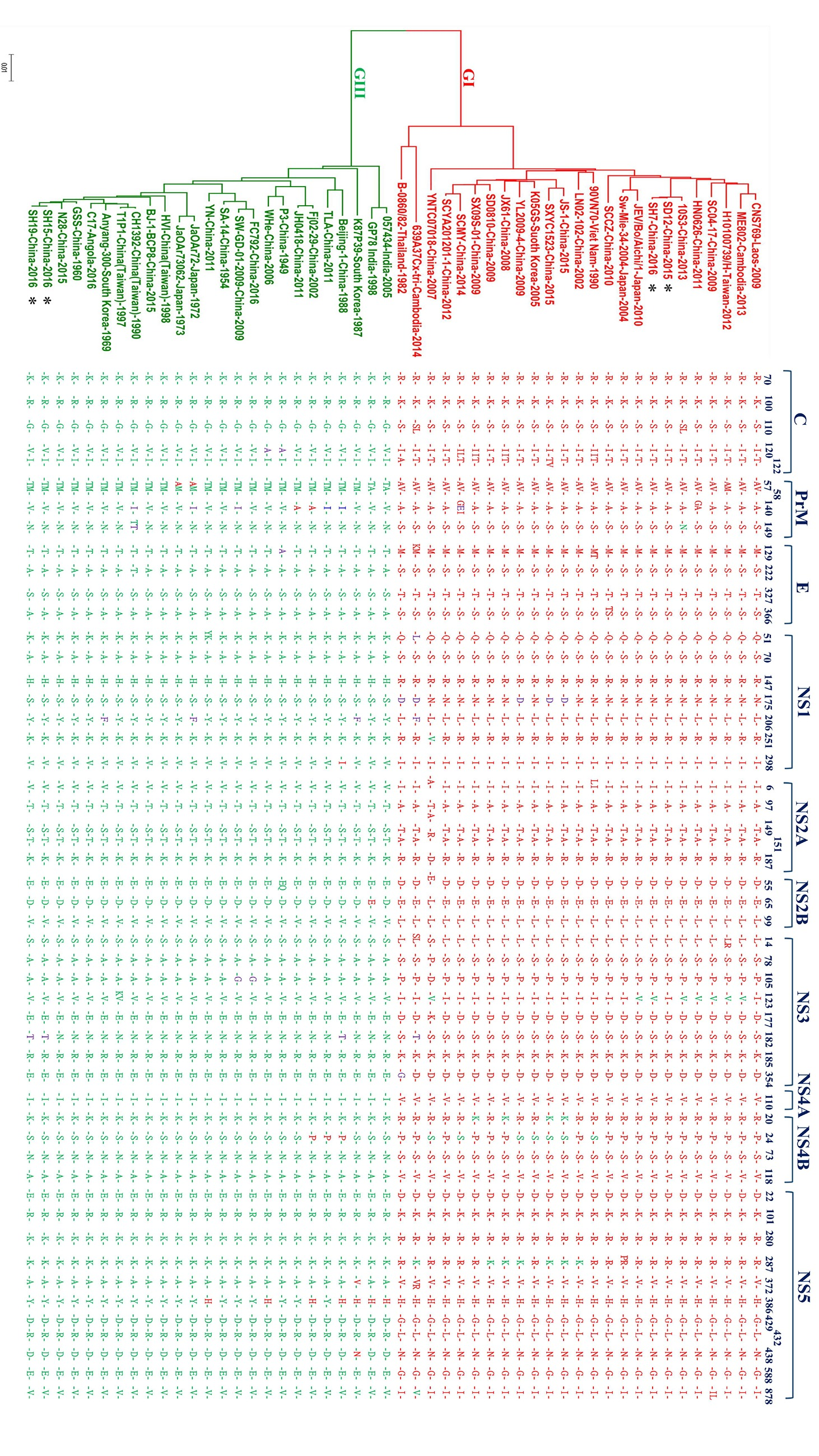

Supplement: S3 Fig — Phylogenetic analysis and multiple sequence alignments were determined with MEGA version 6.06 and DNASTAR software, respectively. The number highlighted in black indicates the amino acid residue position. * indicates the strains used in this study. (TIF) [file ppat.1008773.s003.tif]

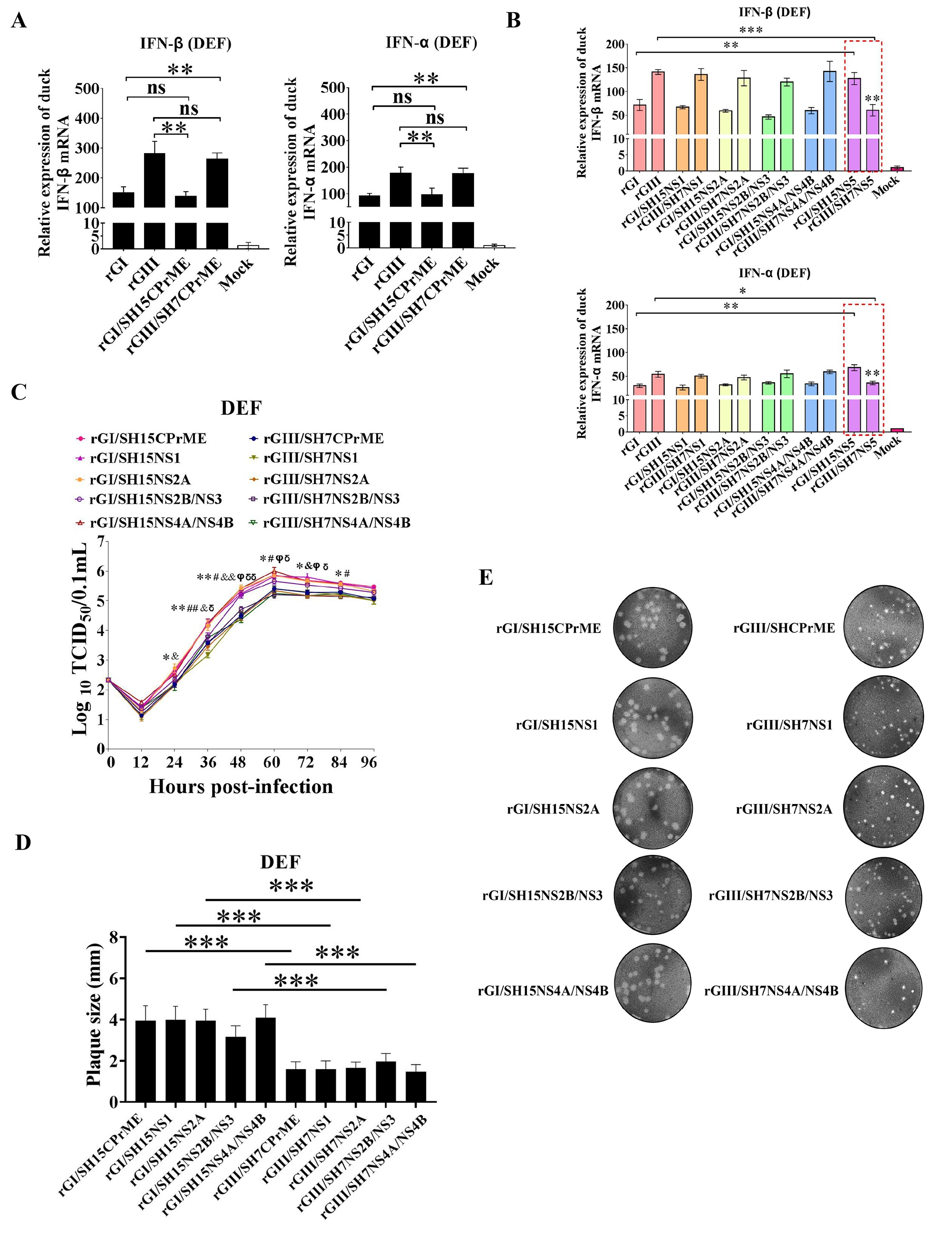

Supplement: S4 Fig — (A and B) DEF were infected with the indicated chimeric recombinant viruses at 1 MOI and harvested at 24 hpi for measurement of IFN-α and β expression at the mRNA level by qRT-PCR. All data are presented as mean ± SD from three independent experiments. *, p < 0.05; **, p < 0.01; ***, p < 0.001; ns, no significant difference, by Student’s t-test. (A) Chimeric recombinant viruses with exchange of structural proteins. (B) Chimeric recombinant viruses with exchange of non-structural proteins. (C) DEF were infected with the indicated chimeric recombinant viruses at 0.01 MOI and harvested at the indicated time points for measurement of viral replication with TCID50 assays in BHK cells. All data are presented as mean ± SD from three independent experiments and were tested by Student’s t-test. The significant difference between rGI/SH15CPrME and rGIII/SH7CPrME at different time points is marked (*, p < 0.05; **, p <0.01). The significant difference between rGI/SH15NS1 and rGIII/SH7NS1 at different time points is labeled (#, p < 0.05; ##, p < 0.01). The significant difference between rGI/SH15NS2A and rGIII/SH7NS2A at different time points is labeled (&, p < 0.05; &&, p < 0.01). The significant difference between rGI/SH15NS2B/NS3 and rGIII/SH7NS2B/NS3 at different time points is labeled (φ, p < 0.05). The significant difference between rGI/SH15NS4A and rGIII/SH7NS4A at different time points is labeled (δ, p < 0.05; δδ, p < 0.01). (D and E) Monolayers of DEF were infected with the indicated chimeric viruses and the respective parental viruses at 100 PFU for analysis of plaque morphology. The plaques were stained with crystal violet at 4 dpi (E) and the plaque diameters were measured and plotted (D). The significant differences between groups were tested by Student’s t-test (***, p < 0.001). (TIF) [file ppat.1008773.s004.tif]

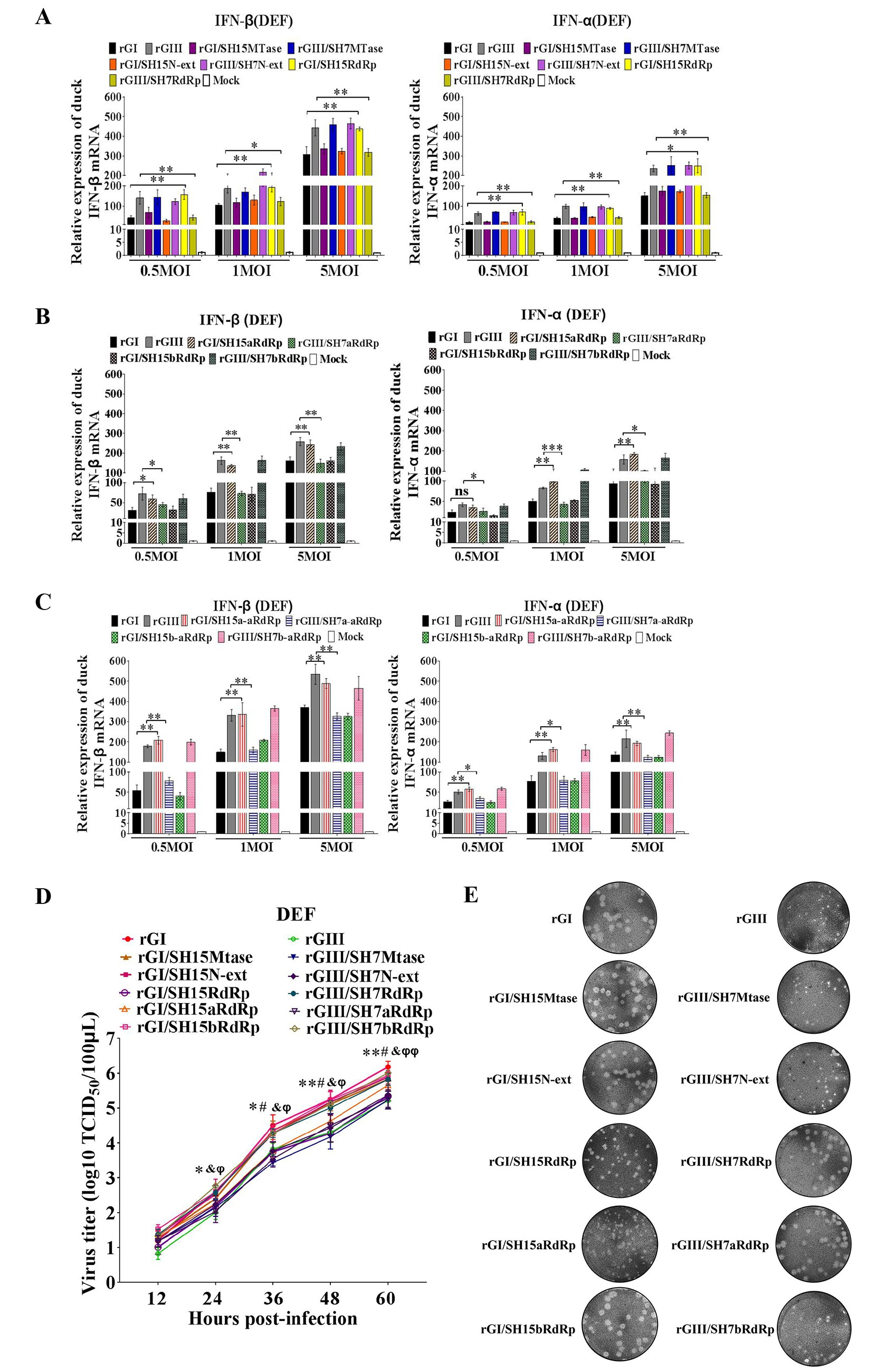

Supplement: S5 Fig — (A, B and C) DEF were infected with the indicated chimeric recombinant viruses at 0.5, 1, and 5 MOI and harvested at 24 hpi for measurement of IFN-α and β expression at the mRNA level by qRT-PCR. All data are presented as mean ± SD from three independent experiments. *, p < 0.05; **, p < 0.01; ***, p <0.001; ns, no significant difference, by Student’s t-test. (A) Chimeric recombinant viruses with exchange of MTase, N-ext, and RdRp. (B) Chimeric recombinant viruses with exchange of aRdRp and bRdRp. (C) Chimeric recombinant viruses with exchange of a-aRdRp and b-aRdRp. (D) DEF were infected with the indicated chimeric recombinant viruses at 0.01 MOI and harvested at the indicated time points for measurement of viral replication with TCID50 assays in BHK cells. All data are presented as mean ± SD from three independent experiments and were tested by Student’s t-test. The significant difference between rGI and rGI/SH15RdRp at different time points is marked (*, p < 0.05; **, p <0.01). The significant difference between rGI and rGI/SH15aRdRp at different time points is labeled (#, p < 0.05).The significant difference between rGIII and rGIII/SH7RdRp at different time points is labeled (&, p < 0.05).The significant difference between rGIII and rGIII/SH7aRdRp at different time points is labeled (φφ, p < 0.01; φ, p < 0.05). (E) Monolayers of DEF were infected with the indicated chimeric viruses and the respective parental viruses at 100 PFU for analysis of plaque morphology. The plaques were stained with crystal violet at 4 dpi. (TIF) [file ppat.1008773.s005.tif]

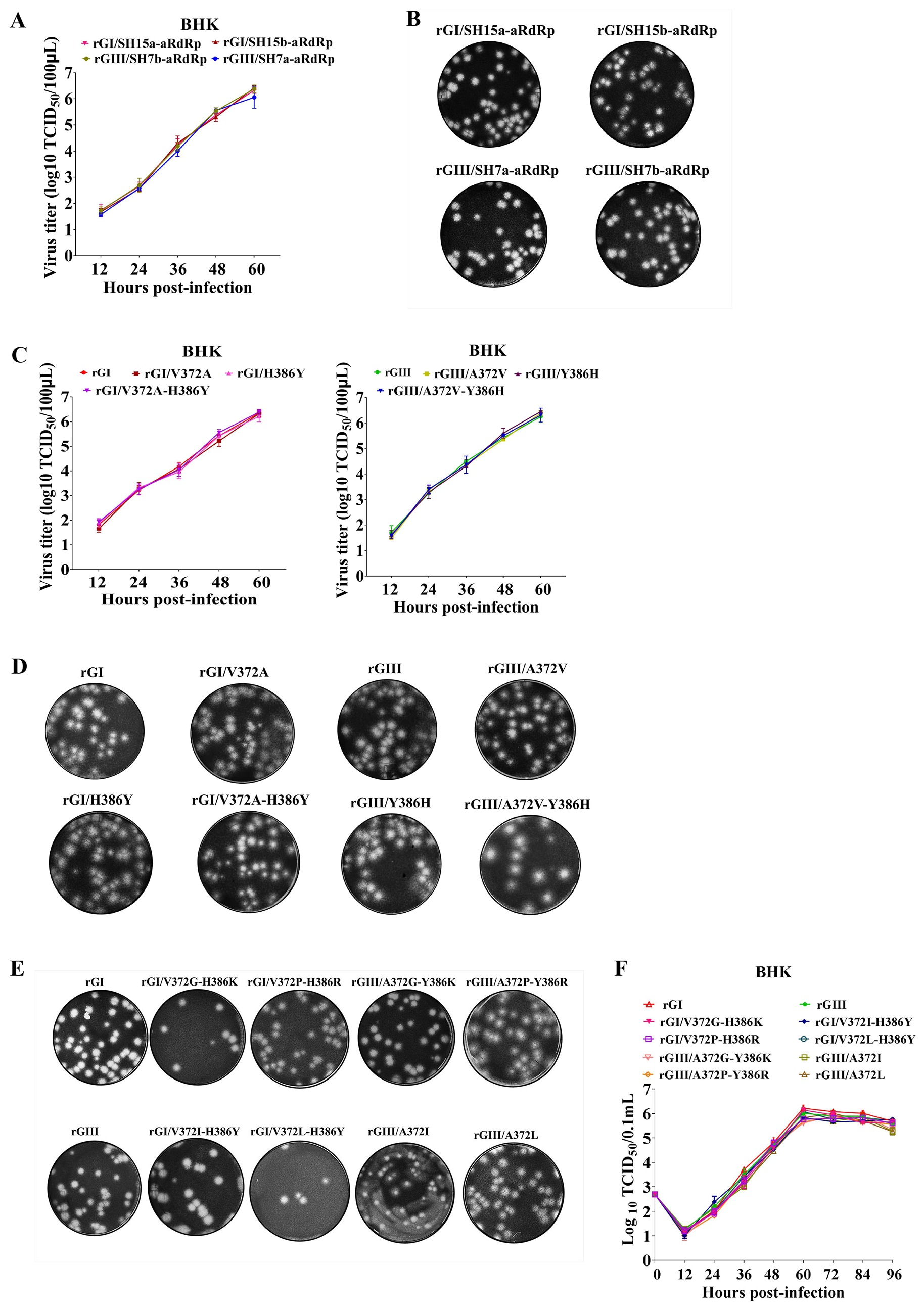

Supplement: S6 Fig — (A, C, and F) BHK cells were infected with the indicated chimeric viruses at 0.01 MOI for analysis of replication efficiency. The supernatants were sampled at the indicated time points and titrated with TCID50 assays on BHK cells. (B, D, and E) Monolayers of BHK cells were infected with the indicated chimeric viruses and the respective parental viruses at 100 PFU for analysis of plaque morphology. The plaques were stained with crystal violet at 4 dpi. (TIF) [file ppat.1008773.s006.tif]

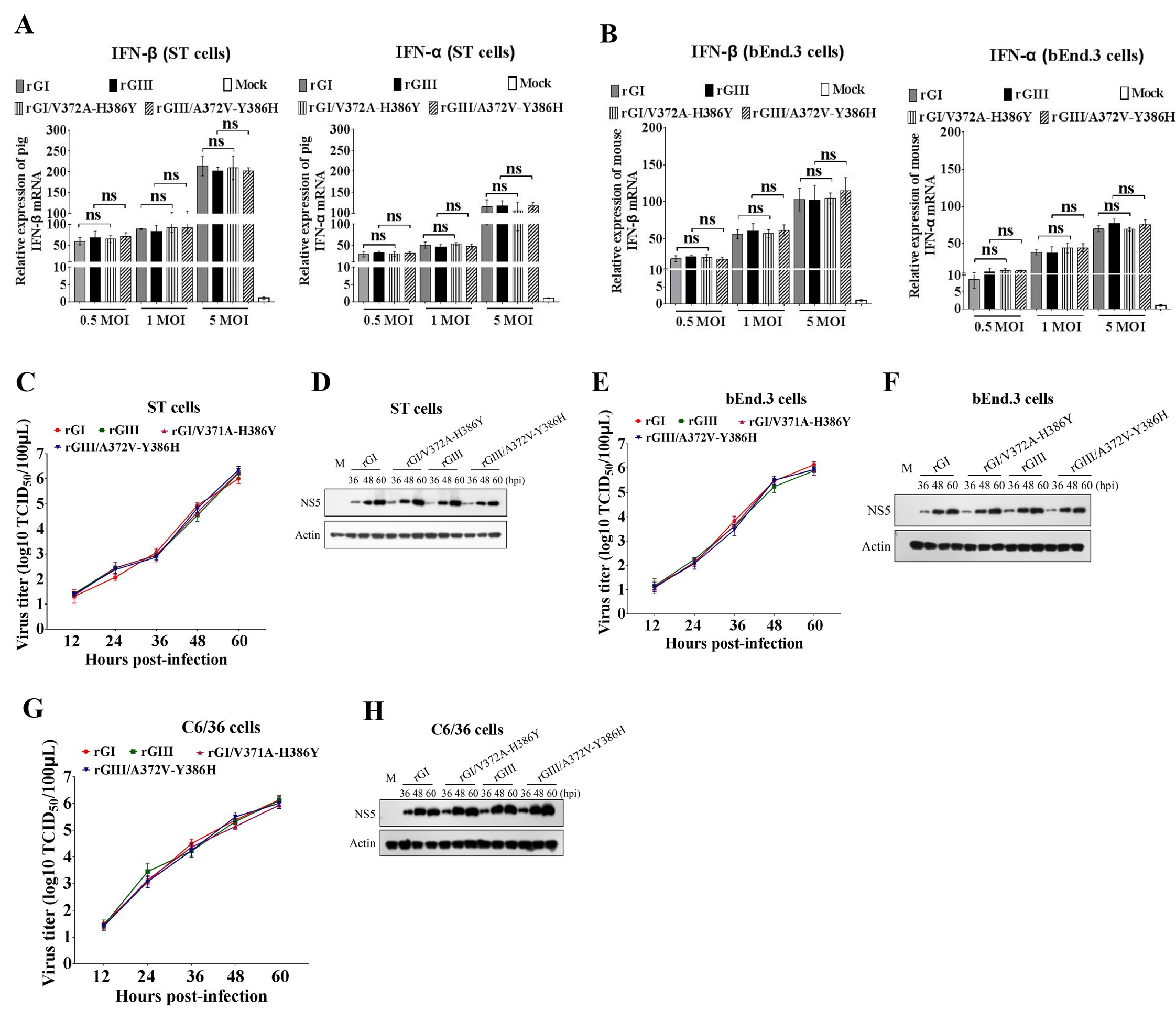

Supplement: S7 Fig — (A and B) ST and bEnd.3 cells were infected with the indicated recombinant viruses at 0.1, 1, and 5 MOI and harvested at 24 hpi for measurement of IFN-α and β production with qR-PCR. (C to H) ST, bEnd.3, and C6/36 cells were infected with the indicated recombinant viruses at a MOI of 0.01 and harvested at the indicated time points for analysis of replication efficiency. The replication titers in the supernatants were titrated with TCID50 assays in BHK cells (C, E and G). The levels of NS5 protein in the cells were examined with western blotting with anti-NS5 antibodies (D, F and H). All data are presented as mean ± SD from three independent experiments. ns, no significant difference by Student’s t-test. (TIF) [file ppat.1008773.s007.tif]
