# Supplementary material for: NS5-V372A and NS5-H386Y variations are responsible for differences in interferon α/β induction and co-contribute to the replication advantage of Japanese encephalitis virus genotype I over genotype III in ducklings
Source: PLoS Pathog. 2020 Sep 3;16(9):e1008773. doi: 10.1371/journal.ppat.1008773 (PMC7494076; doi:10.1371/journal.ppat.1008773)

**1. rGI/V372A-H386Y: GTC (372V) to GCC(372A) and CAC(386H) to TAC (386Y)**


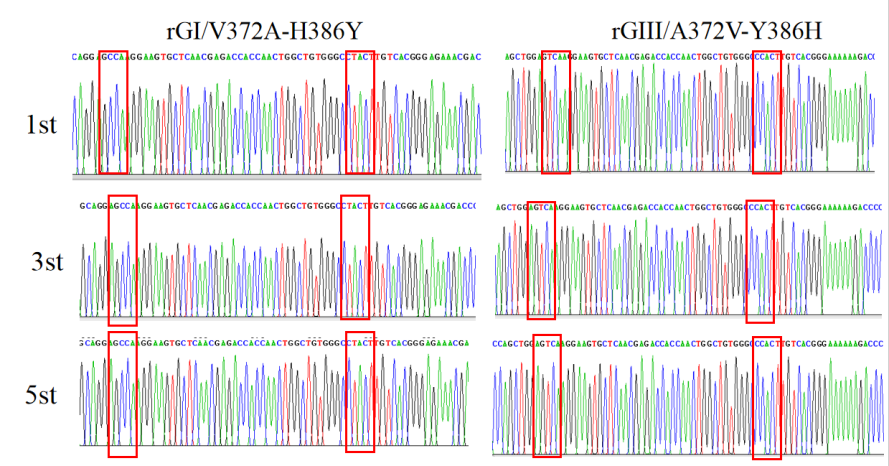


**2. rGIII/A372V-Y386H: GCC (372A) to GTC (372V) and TAC(386Y) to CAC (386H)**


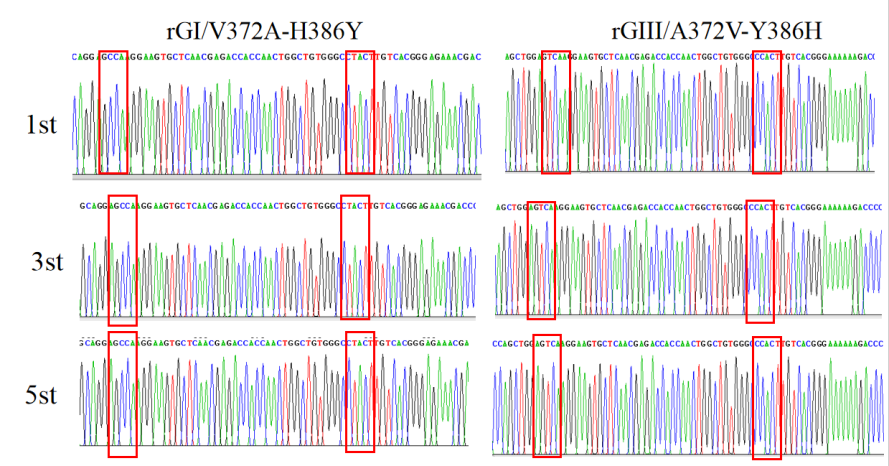


**3. rGI/V372A: GTC (372V) to GCC(372A)**


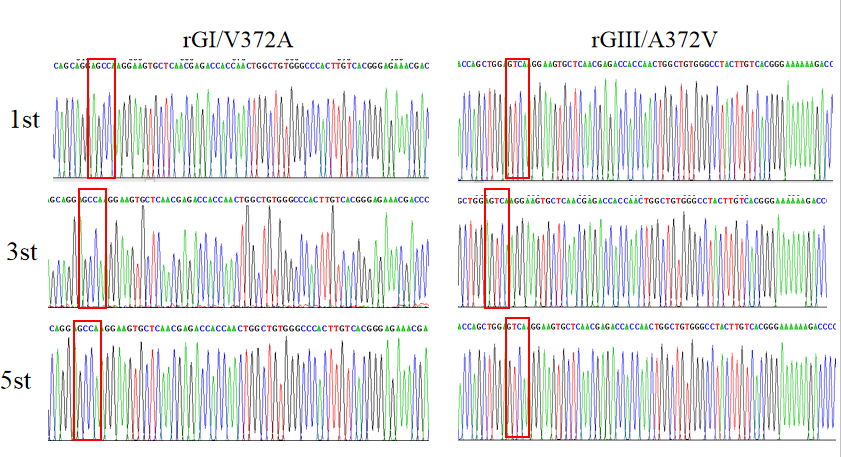


**4. rGIII/A372V: GCC (372A) to GTC (372V)**


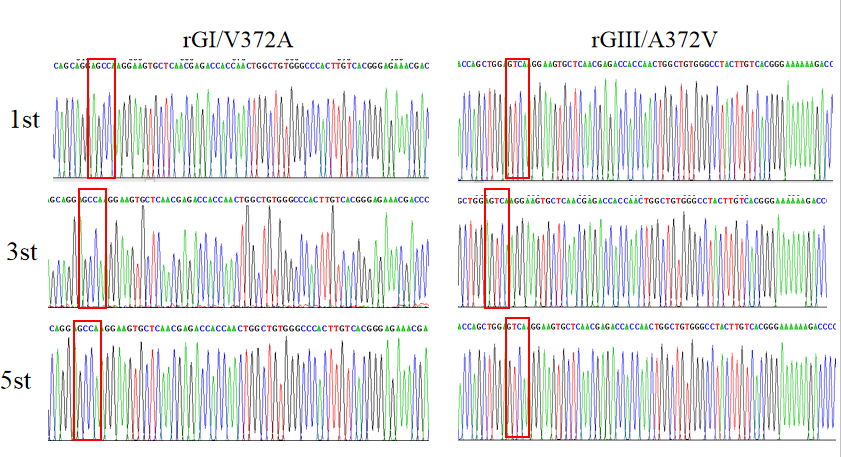


**5. rGI/H386Y: CAC(386H) to TAC (386Y)**


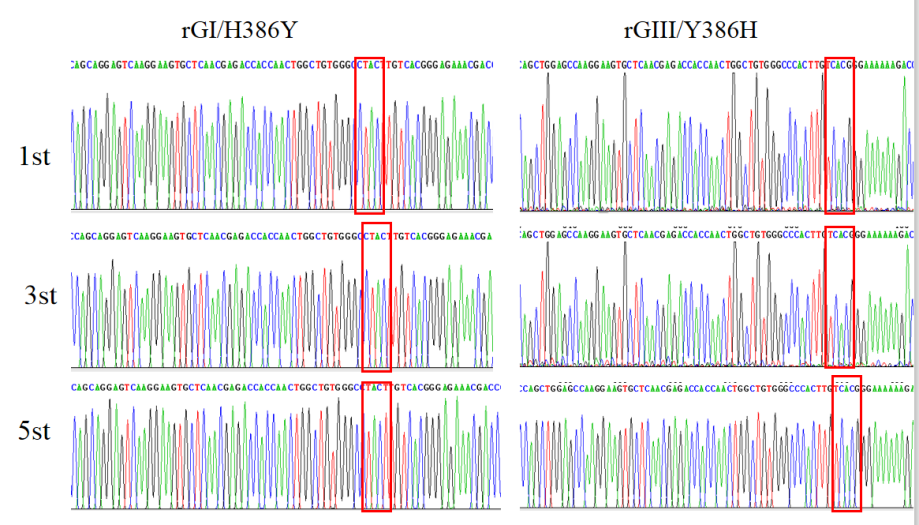


**6. rGIII/Y386H: TAC(386Y) to CAC (386H)**


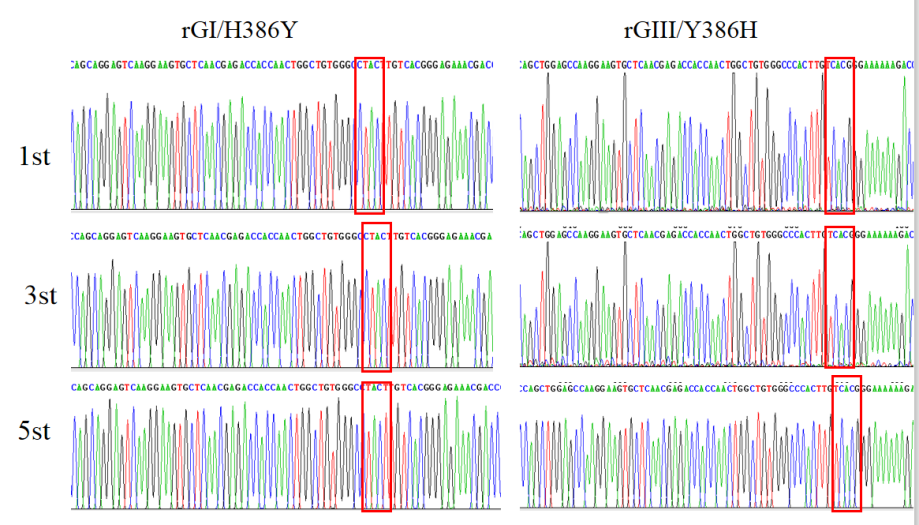


**7. rGI/V372G-H386K: GTC (372V) to GGC (372G) and CAC(386H) to AAA (386K)**


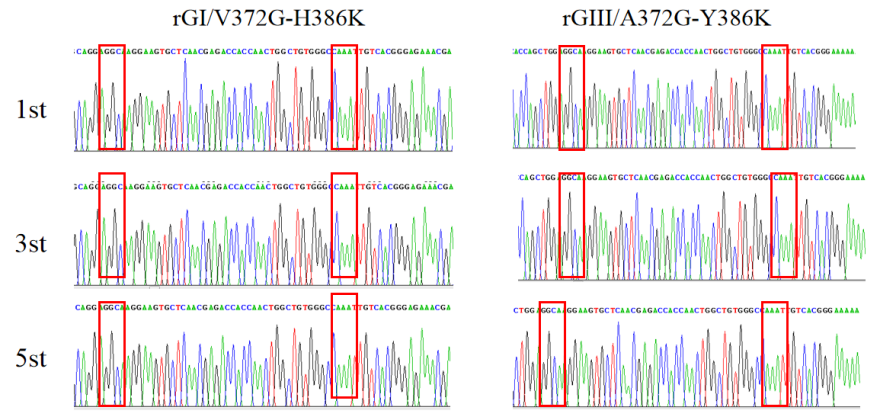


**8. rGIII/A372G-Y386K: GCC (372A) to GGC (372G) and TAC(386Y) to AAA (386K)**


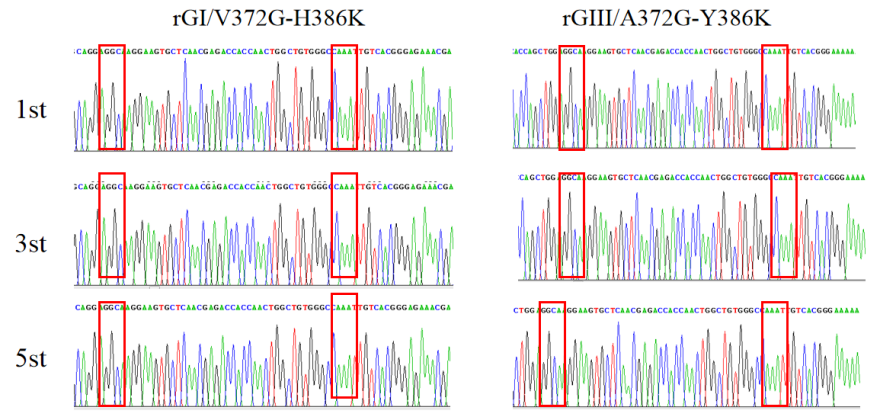


**9. rGI/V372P-H386R: GTC (372V) to CCC (372P) and CAC(386H) to CGC (386R)**


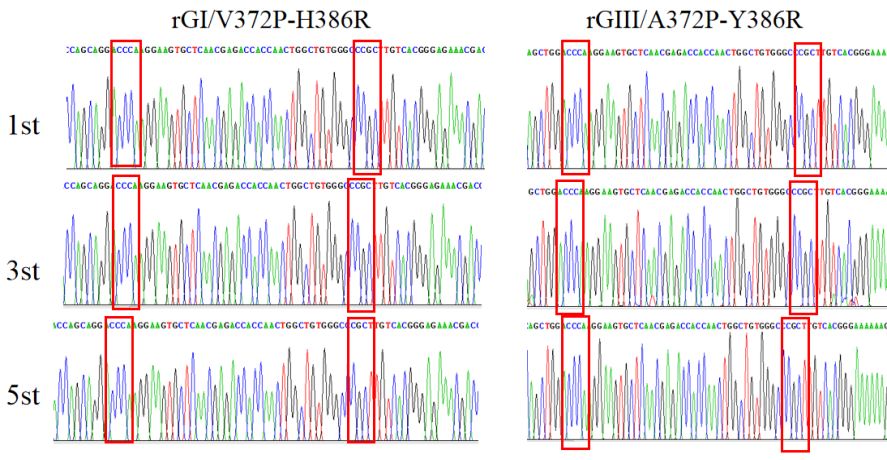


**10. rGIII/A372P-Y386R: GCC (372A) to CCC (372G) and TAC(386Y) to CGC (386K)**


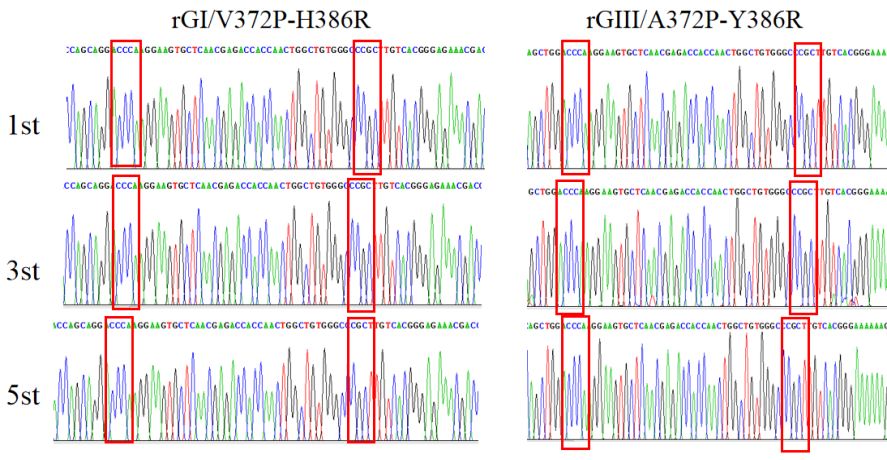


**11. rGI/V372I-H386Y: GTC (372V) to ATC (372I) and CAC(386H) to TAC (386Y)**


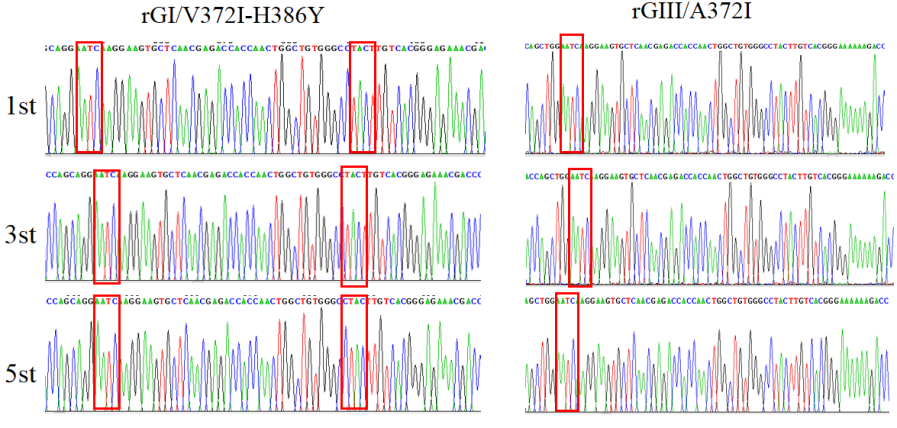


**12. rGIII/A372I: GCC (372A) to ATC (372I)**


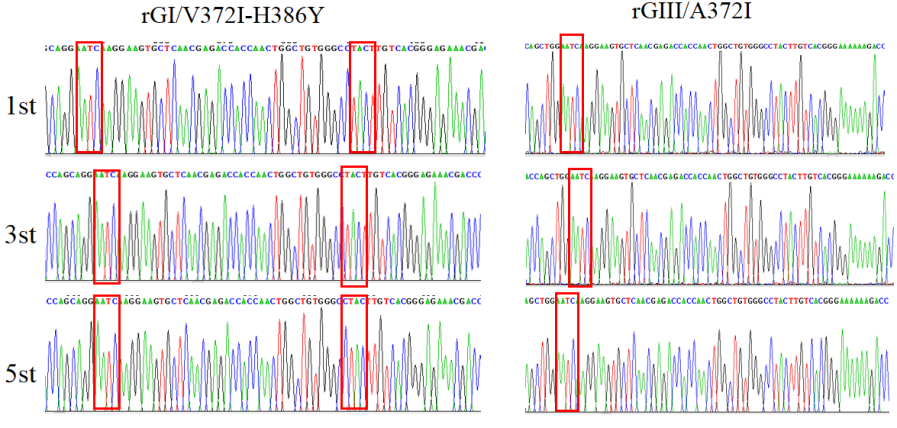


**13. rGI/V372L-H386Y: GTC (372V) to CTC (372I) and CAC(386H) to TAC (386Y)**


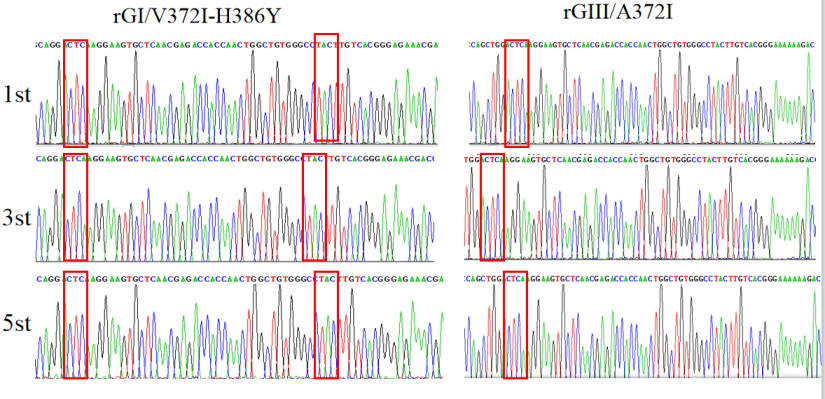


**14. rGIII/A372L: GCC (372A) to CTC (372I)**


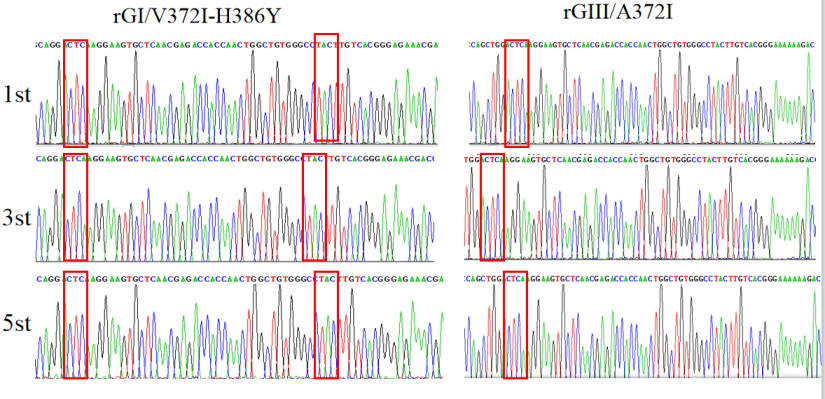

Supplement: S8 Fig — (DOCX) [file ppat.1008773.s008.docx]
