# Supplementary material for: NS5-V372A and NS5-H386Y variations are responsible for differences in interferon α/β induction and co-contribute to the replication advantage of Japanese encephalitis virus genotype I over genotype III in ducklings
Source: PLoS Pathog. 2020 Sep 3;16(9):e1008773. doi: 10.1371/journal.ppat.1008773 (PMC7494076; doi:10.1371/journal.ppat.1008773)
Supplement: S2 Table — (DOCX) [file ppat.1008773.s010.docx]

**S2 Table. Number of hydrogen bonds formed by NS5-372 and NS5-386 with neighboring residues**

| Amino acid | Group | Number of hydrogen bonds formed | |  |
| --- | --- | --- | --- | --- |
|  |  | NS5-372 | NS5-386 |  |
| Val (V) | Nonpolar | 2 | 1 | GI |
| Gly (G) | Nonpolar | 2 | 1 | To replace V or A at NS5-372 |
| Pro (P) | Nonpolar | 1 | 1 | To replace V or A at NS5-372 |
| Phe (F) | Nonpolar | 2 | 1 |  |
| Ala (A) | Nonpolar | 3 | 1 | GIII |
| Ile (I) | Nonpolar | 3 | 1 | To replace V or A at NS5-372 |
| Leu (L) | Nonpolar | 3 | 1 | To replace V or A at NS5-372 |
| Trp (W) | Nonpolar | 3 | 1 |  |
| Met (M) | Nonpolar | 3 | 1 |  |
| His (H) | Polar Basic | 3 | 1 | GI |
| Arg (R) | Polar Basic | 2 | 1 | To replace H or Y at NS5-386 |
| Lys (K) | Polar Basic | 2 | 1 | To replace H or Y at NS5-386 |
| Tyr (Y) | Polar | 2 | 2 | GIII |
| Asn (N) | Polar | 3 | 1 |  |
| Gln (Q) | Polar | 2 | 1 |  |
| Cys (C) | Polar | 2 | 1 |  |
| Ser (S) | Polar | 2 | 1 |  |
| Thr (T) | Polar | 2 | 1 |  |
| Asp (D) | Polar Acidic | 3 | 1 |  |
| Glu (E) | Polar Acidic | 3 | 1 |  |
