# Supplementary material for: NS5-V372A and NS5-H386Y variations are responsible for differences in interferon α/β induction and co-contribute to the replication advantage of Japanese encephalitis virus genotype I over genotype III in ducklings
Source: PLoS Pathog. 2020 Sep 3;16(9):e1008773. doi: 10.1371/journal.ppat.1008773 (PMC7494076; doi:10.1371/journal.ppat.1008773)
Supplement: S3 Table — (DOCX) [file ppat.1008773.s011.docx]

**S3 Table. Primers used in the study**

| Primer name | Sequence (5’to 3') | Positions | Application |
| --- | --- | --- | --- |
| T7-1-2361/2360  (I/III)-F | CTCGAGTGTATA**GTCGAC**TAATACGACTCACTATAGAGAAGTTTATCTGTGTGAACTTCT | 1-24(I/III) | Amplifying SH7 and SH15 |
| T7-1-2361/2360  (I/III)-R | AAACGGCTTTG**GCGGCCGC**TTGTGTGATCCAAGACATTCCCCCAAAGAG | 2332-2361/  2331-2360(I/III) | Amplifying SH7 and SH15 |
| 2332-4497/2331-4496  (I/III)-F | CTCGAGTGTATA**GTCGAC**CTCTTTGGGGGAATGTCTTGGATCACACAA | 2332-2361/  2331-2360(I/III) | Amplifying SH7 and SH15 |
| 2332-4497/2331-4496  (I/III)-R | AAACGGCTTTG**GCGGCCGC**TGGAACACCGGGATCATCAATCAAGTGAAA | 4468-4497/  4467-4496(I/III) | Amplifying SH7 and SH15 |
| 4468-7664/4467-7663  (I/III)-F | CTCGAGTGTATA**GTCGAC**TTTCACTTGATTGATGATCCCGGTGTTCCA | 4468-4497/  4467-4496(I/III) | Amplifying SH7 and SH15 |
| 4468-7664/4467-7663  (I/III)-R | AAACGGCTTTG**GCGGCCGC**GGCTTGTCAGCGTTCTTGATGAGAGTCCA | 7636-7664/  7635-7663(I/III) | Amplifying SH7 and SH15 |
| 7636-10965/7635-10977  (I/III)-F | CTCGAGTGTATA**GTCGAC**TGGACTCTCATCAAGAACGCTGACAAGCC | 7636-7664/  7635-7663(I/III) | Amplifying SH7 and SH15 |
| 7636-10965/7635-10977  (I/III)-R | AAACGGCTTTG**GCGGCCGC**AGATCCTGTGTTCTTCCTCACCACCAG | 10939-10965/  10951-10977(I/III) | Amplifying SH7 and SH15 |
| CPrME-F | GGCATCTTCGTGCACAACGACG | 2530-2551/  2590-2550(I/III) | Structural protein substitution |
| CPrME-R | CGTCGTTGTGCACGAAGATGCC | 2530-2551/  2590-2550(I/III) | Structural protein substitution |
| NS1-F | GGAAATCAGACCTGTTAGGCATGATGAAACAACACT | 3477-3512/  3478-3511(I/III) | NS1 protein substitution |
| NS1-R | AGTATTGTTTCATCATGCCTAACAGGTCTGATTTCC | 3477-3512/  3478-3511(I/III) | NS1 protein substitution |
| NS2A-F | GCCGGACTAATGGTCTGCAACCCAAACAAG | 4180-4219/  4179-4218(I/III) | NS2A protein substitution |
| NS2A-R | CTTGTTTGGGTTGCAGACCATTAGTCCGGC | 4180-4219/  4179-4218(I/III) | NS2A protein substitution |
| NS2B-F | ATGCCTGAGCATTTCATGGGAAAGAC | 6499-6524/  6498-6523(I/III) | NS2B/NS3 protein substitution |
| NS2B-R | GTCTTTCCCATGAAATGCTCAGGCAT | 6499-6524/  6498-6523(I/III) | NS2B/NS3 protein substitution |
| NS4A-F | GAAAAACAGAGGTCACAGACAGA | 6833-6854/  6832-6853(I/III) | NS4A/NS4B protein substitution |
| NS4A-R | TCTGTCTGTGACCTCTGTTTTTC | 6833-6854/  6832-6853(I/III) | NS4A/NS4B protein substitution |
| NS5-F | TTGATCCAGGA**R**GA**Y**AGGGTCAT | 10369-10391/  10368-10390(I/III) | NS5/NS5RdRp domain protein substitution |
| NS5-R | ATGACCCT**R**TC**Y**TCCTGGATCAA | 10369-10391/  10368-10390(I/III) | NS5/NS5RdRp domain protein substitution |
| NS5-Mtase-F | GGATCGCACAGTGTGGAGAGGGCC | 8409-8432/  8408-8431(I/III) | Mtase domain substitution |
| NS5-Mtase-R | GGCCCTCTCCACACTGTGCGATCC | 8409-8432/  8408-8431(I/III) | Mtase domain substitution |
| NS5-N-ext-F | ATGGCCATGACTGACACCACCCC | 8704-8726/  8703-8725(I/III) | N-ext domain substitution |
| NS5-N-ext-R | GGGGTGGTGTCAGTCATGGCCAT | 8704-8726/  8703-8725(I/III) | N-ext domain substitution |
| NS5-a/bRdRp-F | CCATTGGCTGAGCCGAGAGAATTCAGG | 9168-9194/  9167-9193(I/III) | a/bRdRp domain substitution |
| NS5-a/bRdRp-F | CAAGGAAGTGCTCAACGAGACCACCAAC | 9168-9194/  9167-9193(I/III) | a/bRdRp domain substitution |
| NS5-a/b-aRdRp-F | CAAGGAAGTGCTCAACGAGACCACC | 8793-8817/  8792-8816(I/III) | a/b-aRdRp domain substitution |
| NS5-a/b-aRdRp-F | GGTGGTCTCGTTGAGCACTTCCTTG | 8793-8817/  8792-8816(I/III) | a/b-aRdRp domain substitution |
| GI-NS5-V372A-F | CACCAGCAGGAGCCAAGGAAGTGCTC | 8780-8805(I) | NS5 V372A mutation |
| GI-NS5-V372A-R | GAGCACTTCCTTGGCTCCTGCTGGTG | 8780-8805(I) | NS5 V372A mutation |
| GIII-NS5-A372V-F | CACCAGCTGGAGTCAAGGAAGTGCTC | 8779-8804(III) | NS5 A372V mutation |
| GIII-NS5-A372V-R | GAGCACTTCCTTGACTCCAGCTGGTG | 8779-8804(III) | NS5 A372V mutation |
| GI-NS5-H386Y-F | ACTGGCTGTGGGCCTACTTGTCACGGG | 8819-8845(I) | NS5 H386Y mutation |
| GI-NS5-H386Y-R | CCCGTGACAAGTAGGCCCACAGCCAGT | 8819-8845(I) | NS5 H386Y mutation |
| GIII-NS5-Y386H-F | ACTGGCTGTGGGCCCACTTGTCACGGG | 8818-8844(III) | NS5 Y386H mutation |
| GIII-NS5-Y386H-R | CCCGTGACAAGTGGGCCCACAGCCAGT | 8818-8844(III) | NS5 Y386H mutation |
| GI-NS5-△372V-F | GCCACCAGCAGGAAAGGAAGTGCTCAA | 8778-8807(I) | NS5 V372 deletion |
| GI-NS5-△372V-R | TTGAGCACTTCCTTTCCTGCTGGTGGC | 8778-8807(I) | NS5 V372 deletion |
| GIII-NS5-△372A-F | GCCACCAGCTGGAAAGGAAGTGCTCAA | 8777-8806(III) | NS5 372A deletion |
| GIII-NS5-△372A-R | TTGAGCACTTCCTTTCCAGCTGGTGGC | 8777-8806(III) | NS5 372A deletion |
| GI/III-NS5-△386-F | TCCCGTGACAAGGCCCACAGCC | 8823-8846/  8822-8865(I/III) | NS5 386H/Y deletion |
| GI/III-NS5-△386-R | GGCTGTGGGCCTTGTCACGGGA | 8823-8846/  8822-8865(I/III) | NS5 386H/Y deletion |
| NS5-372G-F | GCCACCAGCWGGA**GGC**AAGGAAGTG | 8778-8802/  8776-8801(I/III) | NS5 372G mutation |
| NS5-372G-R | CACTTCCTT**GCC**TCCWGCTGGTGGC | 8778-8802/  8776-8801(I/III) | NS5 372G mutation |
| NS5-372P-F | GCCACCAGCWGGA**CCC**AAGGAAGTG | 8778-8802/  8776-8801(I/III) | NS5 372P mutation |
| NS5-372P-R | CACTTCCTT**GGG**TCCWGCTGGTGGC | 8778-8802/  8776-8801(I/III) | NS5 372P mutation |
| NS5-372I-F | GCCACCAGCWGGA**ATC**AAGGAAGTG | 8778-8802/  8776-8801(I/III) | NS5 372I mutation |
| NS5-372I-R | CACTTCCTT**GAT**TCCWGCTGGTGGC | 8778-8802/  8776-8801(I/III) | NS5 372I mutation |
| NS5-372L-F | GCCACCAGCWGGA**CTC**AAGGAAGTG | 8778-8802/  8776-8801(I/III) | NS5 372L mutation |
| NS5-372L-R | CACTTCCTT**GAG**TCCWGCTGGTGGC | 8778-8802/  8776-8801(I/III) | NS5 372L mutation |
| NS5-386K-F | GGCTGTGGGCC**AAA**TTGTCACGGGA | 8823-8846/  8822-8865(I/III) | NS5 386K mutation |
| NS5-386K-R | TCCCGTGACAA**TTT**GGCCCACAGCC | 8823-8846/  8822-8865(I/III) | NS5 386K mutation |
| NS5-386R-F | GGCTGTGGGCC**CGC**TTGTCACGGGA | 8823-8846/  8822-8865(I/III) | NS5 386R mutation |
| NS5-386R-R | TCCCGTGACAA**GCG**GGCCCACAGCC | 8823-8846/  8822-8865(I/III) | NS5 386R mutation |
| qduIFN-β-F | TTCACCTCAGCATCAACA | _ | qRT-PCR primer |
| qduIFN-β-R | TCTTCATCCGCCGTATTAG | _ | qRT-PCR primer |
| qduIFN-α-F | CCTCCTCCAACACCTCTT | _ | qRT-PCR primer |
| qduIFN-α-R | GTGGATGTGGTGCTGAAG | _ | qRT-PCR primer |
| qduGAPDH-F | GATGCTGGTGCTGAATAC | _ | qRT-PCR primer |
| qduGAPDH-R | GAGATGATGACACGCTTAG | _ | qRT-PCR primer |
| qPig-IFN-β-F | ATCCTCCAAATCGCTCTCCT | _ | qRT-PCR primer |
| qPig-IFN-β-R | TTGTGGTGGTTGCATAATCTC | _ | qRT-PCR primer |
| qPig-IFN-α-F | AGAAGGATGATCGACTCA | _ | qRT-PCR primer |
| qPig-IFN-α-R | AAATAGACCTGCCCAGAT | _ | qRT-PCR primer |
| qPigGAPDH-F | TCTGGCAAAGTGGACATT | _ | qRT-PCR primer |
| qPigGAPDH-R | GGTGGAATCATACTGGAACA | _ | qRT-PCR primer |
| qMouse-IFN-β-F | GCTGCGTTCCTGCTGTGCT | _ | qRT-PCR primer |
| qMouse-IFN-β-R | CATCTTCTCCGTCATCTCCA | _ | qRT-PCR primer |
| qMouse-IFN-α-F | CCCGCAGGAGAAGGTGGAT | _ | qRT-PCR primer |
| qMouse-IFN-α-R | GAGCTGCTGGTGGAGGTCA | _ | qRT-PCR primer |
| qMouseGAPDH-F | GAGGCCGGTGCTGAGTATGT | _ | qRT-PCR primer |
| qMouseGAPDH-R | CGGCAGAAGGGGCGGAGATG | _ | qRT-PCR primer |
